# Realizations of the triple points of carbon dioxide and sulfur hexafluoride—ITS-90 temperature determinations and interpolations

**DOI:** 10.1088/1681-7575/ae81ba

**Published:** 2026-08-05

**Authors:** Klaus N Quelhas, Richmond S Wang, Andrea Peruzzi, Weston L Tew

**Affiliations:** 1 Theiss Research, La Jolla, California, United States of America; 2 Physical Measurement Laboratory, National Institute of Standards and Technology, Gaithersburg, Maryland, United States of America; 3 National Research Council of Canada, Ottawa, Canada

**Keywords:** carbon dioxide, sulfur hexafluoride, mercury, triple point, ITS-90, temperature scales, standard platinum resistance thermometer

## Abstract

The triple points of carbon dioxide (TP CO_2_) and sulfur hexafluoride (TP SF_6_) have been studied over the last decade as alternative fixed points to the triple point of mercury (TP Hg, 234.3156 K) on the International Temperature Scale of 1990 (ITS-90). NIST has been developing TP CO_2_ and TP SF_6_ cells to be employed as drop-in replacements for TP Hg cells for calibrations of standard platinum resistance thermometers (SPRTs). This work presents the results of the assessment of new immersion-type CO_2_ and SF_6_ triple point cells built at NIST and the results of their use in ITS-90 calibrations of long-stem SPRTs. It was demonstrated that a very well-controlled realization technique can result in measurements of the triple points of CO_2_ and SF_6_ within 25 μK and 75 μK of reproducibility, respectively, with estimated temperatures of 216.591 21(15) K and 223.556 22(33) K for *F*= 1, respectively. The large volume expansion of CO_2_ and SF_6_ upon melting in large immersion cells is responsible for a progressive head correction that increases the realization slopes of the triple points of CO_2_ and SF_6_ by 0.1 mK and 0.46 mK, respectively. Calibrations of two SPRTs on different ITS-90 subranges using either TP Hg, TP CO_2_ or TP SF_6_ cells showed that the interpolated temperatures using either triple point agreed within their realization uncertainties. These results demonstrate that both fixed points can be used as alternatives for the Hg TP in a future revision of the ITS-90.

## 1. Introduction

Increasing restrictions on the manufacture and sale of products containing mercury, like the U.S. Mercury Export Ban Act (2008) and the Minamata Convention on Mercury (2013, entered into force in 2017) motivated a group of National Metrology Institutes (NMIs) to investigate potential replacements for the triple point of mercury (TP Hg, 234.3156 K) in the International Temperature Scale of 1990 (ITS-90) [1] for the calibration of Standard Platinum Resistance Thermometers (SPRTs) [2]. Currently there are three candidates: the triple points of carbon dioxide (TP CO_2_, 216.591 K), sulfur hexafluoride (TP SF_6_, 223.556 K) and xenon (TP Xe, 161.406 K) [3]. While the first two can be realized in low temperature stirred liquid baths currently used for the TP Hg, the lower temperature of the TP Xe requires a special cryostat for realization.

Recent investigations on the TP CO_2_ include contributions from NMIJ [4], VNIIM [5], NIM [6] and NPL [7], while recent studies on the TP SF_6_ include investigations performed by NRC [8], NIST [2], NMIJ [9], NIM [10] and SMU [11]. Both small cells for calibrating capsule-type SPRTs (CSPRTs) inside adiabatic calorimeters and large immersion cells for calibrating long-stem SPRTs (LSPRTs) were investigated in these studies. The construction of immersion-type cells is of greater complexity due to the handling of large volume cells containing gases at high pressures, especially for CO_2_ cells, and the need to achieve adequate immersion in the presence of heat leaks along the LSPRT stems.

As part of its investigation of the triple points of SF_6_ and CO_2_ as potential replacements for the TP Hg used for calibrations of SPRTs, NIST has manufactured two small adiabatic and four immersion SF_6_ cells, as well as two immersion CO_2_ cells. The immersion cells were designed to be employed as drop-in replacements for TP Hg cells, i.e. they were made to be used in conventional low-temperature stirred liquid baths in the same way traditional TP Hg cells are used for the calibration of LSPRTs, ensuring minimal impact on the requirements for commonly installed laboratory equipment.

The results of two adiabatic and two immersion SF_6_ cells at NIST were previously reported in [2], and this work reports on the manufacture and analysis of two immersion CO_2_ cells and one additional immersion SF_6_ cell. We demonstrate how the realization technique has a strong impact on the reproducibility of the measured temperatures of both fixed points. Our technique employs a well-controlled inner freeze method, followed by an inner melt using an immersion heater and a quasi-adiabatic pulsed realization. The results of calibrations of two SPRTs on the subranges 4, from the Ar TP to the triple point of water (TPW) and 5, from the Hg TP to the Ga MP, of the ITS-90 using TP Hg, TP CO_2_ and TP SF_6_ cells is also reported in this work, showing the equivalence between the temperatures interpolated using either fixed point, demonstrating that both TP CO_2_ and TP SF_6_ can be used for regular calibrations of SPRTs.

This work is structured as follows: section 2 describes the design, filling and assessment of the CO_2_ and SF_6_ cells, section 3 describes the use of these cells for the calibration of two SPRTs, section 4 shows the results of the assessment of the cells and the calibration of the SPRTs, section 5 discusses the results found in this study, including analyses of the effects of a progressive head correction, impurity distributions and isotopic variations, the analysis of uncertainties, their propagation, subrange inconsistencies (SRI) and non-uniqueness (NU), and section 6 concludes this work.

## 2. Design and filling the cells

Table 1 provides some relevant information relative to the triple points of CO_2_ and SF_6_, as well as for the TP Hg for comparison purposes [12]. The most striking difference lies in the vapor pressures, which are ∼9 orders of magnitude higher for CO_2_ and SF_6_ in comparison to Hg. This requires the adoption of containers that can withstand high pressures, especially when the cells are stored at room temperature. Additionally, the volume contractions (expansions) of CO_2_ and SF_6_ upon freezing (melting) are significantly larger than that of Hg, with freezing contractions relative to the liquid of 23.1 % and 19 %, respectively, versus 3.5 % of mercury. These large volume changes necessitate special procedures for realizing these fixed points. Finally, even though the latent heats of melting per mass of CO_2_ and SF_6_ are significantly higher than that of Hg, their lower densities result in cells with lower values of enthalpy for the same volume, so the average heating rates are lower for similar plateaus with these cells.

**Table 1.** Some properties of carbon dioxide [13, 14], sulfur hexafluoride [2, 12] and mercury [15] at their triple points. *T*_TP_ and *P*_TP_ are the triple point temperature and pressure, *ρ*_l,TP_, *ρ*_s,TP_, *v*_l_ and *v*_s_ are the liquid and solid densities and specific volumes at the triple point, Δ*h*_f,TP_ is the enthalpy of fusion at the triple point, d*T*_TP_/d*h* is the hydrostatic head correction, and *A^−^*^1^ is the inverse of the first cryoscopic constant.

Substance	*T*_TP_ K	*P*_TP_ MPa	*ρ*_l,TP_ kg m^−3^	*ρ*_s,TP_ kg m^−3^	*(v*_l_*–v*_s_*)/v*_l_ *_%_*	*(v*_l_*–v*_s_*)/v*_s_ *_%_*	Δ*h*_f,TP_ kJ kg^−1^	d*T*_TP_*/*d*h* mK m^−1^	*A^−^*^1^ μK ppm^−1^
CO_2_	216.591	0.518	1178	1532	23.1	30.0	200	2.47	45
SF_6_	223.556	0.231	1845	2279	19.0	23.5	36.15	11.6	79
Hg	234.3156	1.7 × 10^−10^	13 690	14 184	3.5	3.6	11.43	8.6	199

**Figure 1.**
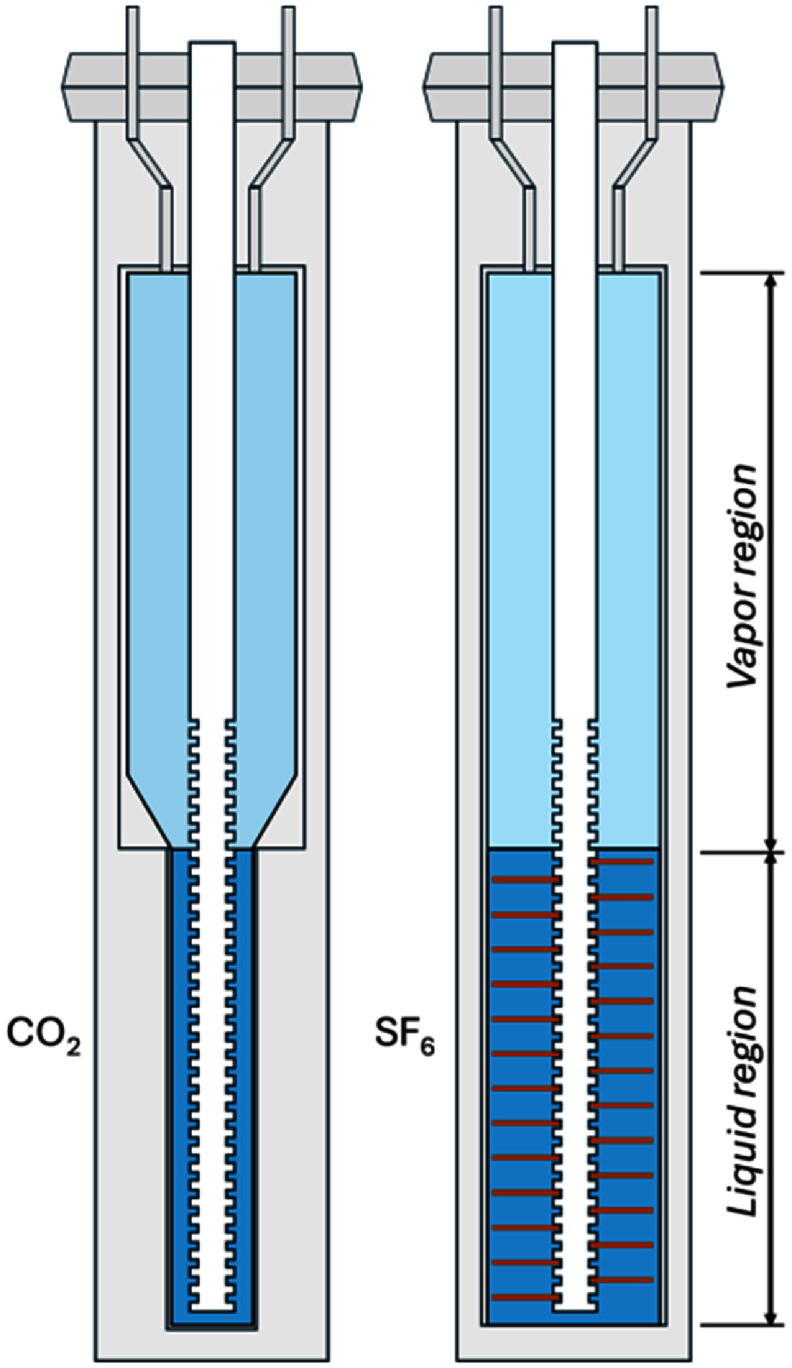
Designs of the TP CO_2_ (left) and TP SF_6_ (right) cells. Drawings not to scale.

With these constraints in mind, the cells were designed as show in figure 1, and made of electropolished 316L stainless steel by the NIST machine shop. The cell containers were designed to hold pressures higher than 14 MPa. The cells were tested using a helium leak detector, followed by a hydrostatic stress test at 7 MPa using distilled water. The cells were then baked at 120 °C under vacuum to remove any residual water and finally tested again with the leak detector. To allow for quasi-adiabatic realizations, they are placed inside insulating jackets under vacuum or filled with a low thermal conductivity gas. Electrical heaters attached to the outside of the liquid region of the cells are connected to a controlled current source to provide quasi-adiabatic pulsed realizations.

**Figure 2.**
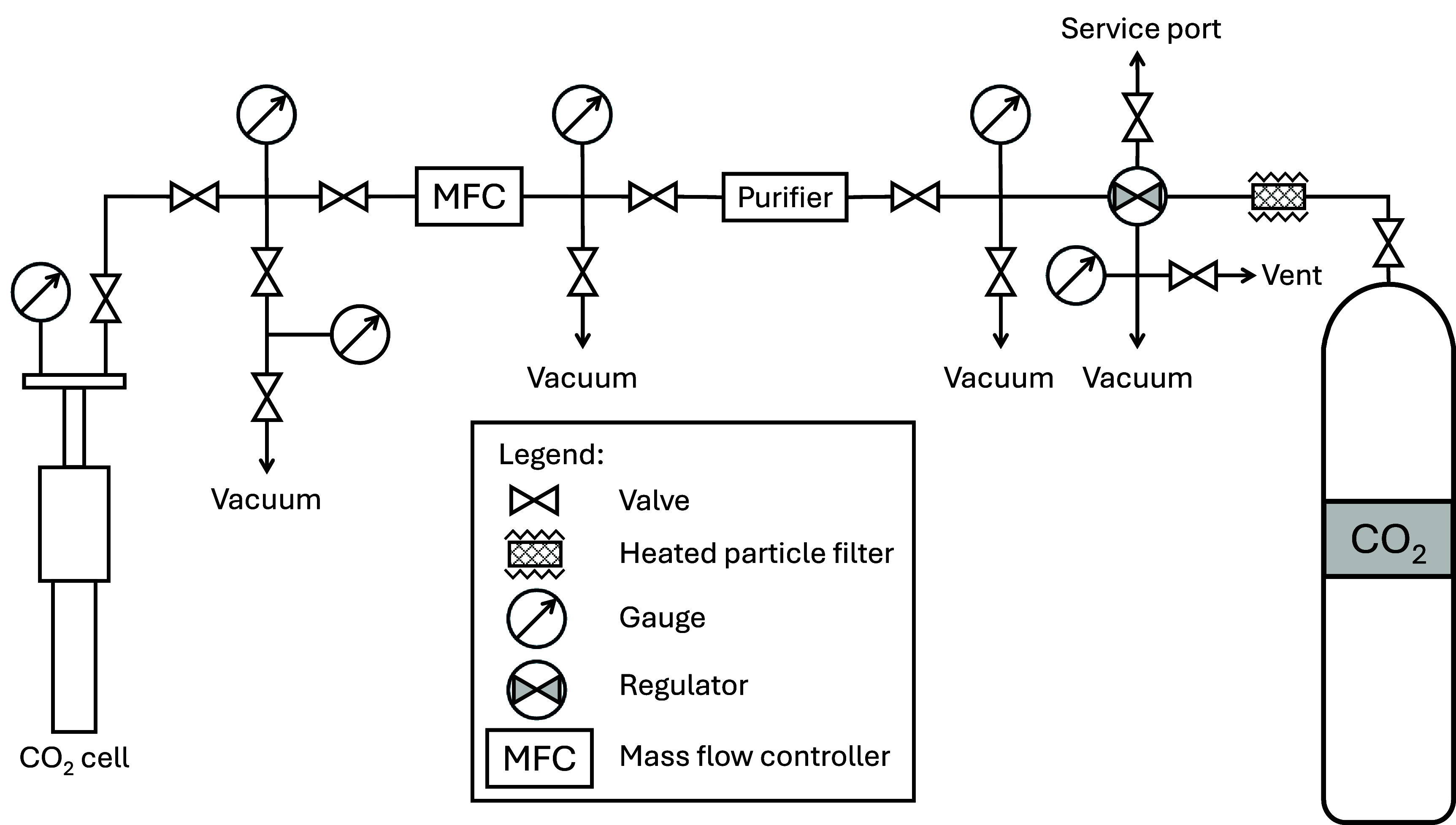
Diagram describing the manifold used to fill cell CO_2_ 811. Drawing not to scale.

The manifold employed to fill the cells is similar to the one previously used in [2]. Referring to figure 2, the pressure of the gas phase flow from the source cylinder is regulated down by a high purity pressure regulator, an in-line purifier containing a passive chemical getter removes reactive gases, moisture, organics and metal vapors to less than 10^−9^ in volume fraction [16], a mass flow controller helps controlling the amount of gas transferred to the cell, and several gauges keep track of the pressures between each stage and at the connection point to the cell. A molecular-drag vacuum pump is connected to each stage, and the whole setup was tested with a helium leak detector before filling each cell. The cells were pumped and flushed with 0.1 MPa of high purity gas at least two times before the final filling. Both cell designs include a 40 mm dial-type bourdon-tube pressure gauge at the measurement port and a spring bellows valve at the input line. The valves are rated for use at pressures up to 7 MPa.

### 2.1. CO_2_ cells

Two TP CO_2_ cells were filled for this study, identified as 810 and 811, and filled in 2019 and 2025, respectively. Both cells have a 25 mm outer diameter (OD) lower liquid region and a 50 mm OD upper region. The smaller lower region in comparison to the upper region is necessary to limit the amount of liquid while maintaining a good SPRT immersion, and to accommodate the gas as it vaporizes, particularly near room temperatures, where the CO_2_ is completely in vapor form and the cell internal pressure can reach over 5.5 MPa, but still below the CO_2_ critical pressure (∼7.4 MPa). The heights of the lower and upper regions are 190 mm and 205 mm, respectively. A 12 mm OD, 8 mm inner diameter (ID) thermowell was welded inside the container to accommodate LSPRTs. The thermowell contains 1 mm (depth) by 2.5 mm (width) grooves on the outer surface to increase the heat exchange area and support the solid CO_2_ mantle. The tare mass of both cells was measured with a precision scale, being around 1.77 kg.

Cell 810 was filled with gas provided by the NIST gas metrology group, with a measured content of nitrogen of 1.05 μmol mol^−1^, and a measured content of argon of 0.30 μmol mol^−1^, with an uncertainty of 0.03 μmol mol^−1^. Cell 811 was filled with 6N minimum purity gas provided by Linde, with a measured content of nitrogen of less than 0.04 μmol mol^−1^, 0.02 μmol mol^−1^ of oxygen, 0.147 μmol mol^−1^ of moisture and a content of hydrocarbons of less than 0.02 μmol mol^−1^. Table 2 provides a summary of the cells studied in this work.

**Table 2.** Summary of triple point cells studied in this work.

Cell ID	Gas	Volume	Gas mass	Source	Year
803	5N5 SF_6_	537 cm^3^	573.3 g	E.F. LLC	2019
810	6N CO_2_^a^	337 cm^3^	68.55 g	NIST GMG	2019
811	6N CO_2_	337 cm^3^	68.03 g	Linde USA	2025

^a^ See text for additional information regarding impurity content.

**Figure 3.**
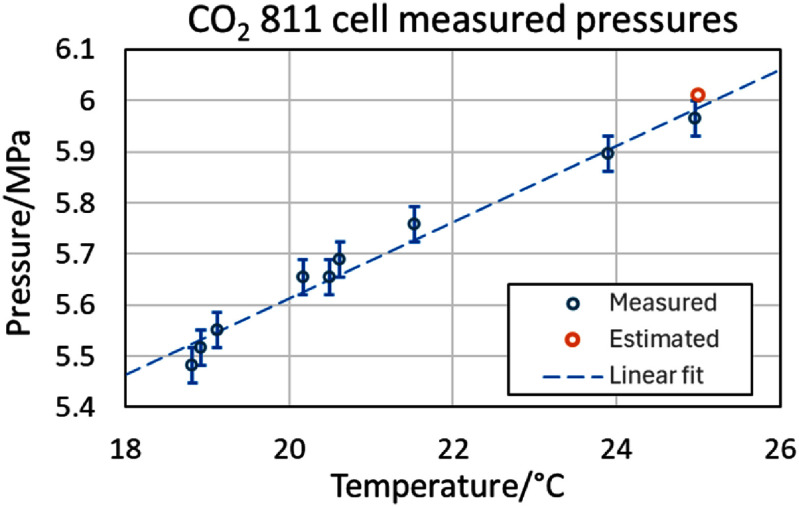
Measured pressures (blue circles) of cell CO_2_ 811 for different room temperatures and the estimated pressure at 25 °C (orange circle). Dashed line corresponds to a linear fit of the measured pressures.

The cells were cooled down to −55 °C and filled at a pressure of 0.55 MPa and a flow of 500 cm^3^ min^−1^ (standard conditions, or ‘sccm’) for ∼1 h. Once each cell was filled, the input valve was closed. An additional step was adopted for cell 810 only; after its filling, the cell was immersed in liquid argon while still connected to the manifold under high vacuum, and once the CO_2_ was frozen the valve was opened, revealing a small partial pressure of volatile gases (presumably most of it was nitrogen), which were pumped away before closing the valve and allowing the cell to warm up to room temperature. Cells 810 and 811 were weighed again and the resulting gas masses were 68.55 g and 68.03 g, respectively. Since the CO_2_ is completely vaporized at room temperature, it was possible to verify the amount of gas by measuring the internal pressures of the cells with their dial pressure gauges. Figure 3 shows the pressures of cell CO_2_ 811 at different room temperatures over several weeks after the cell was filled, and the calculated pressure based on volume estimates and the properties of CO_2_ provided by REFPROP [12]. The measured pressures agree with our estimates to less than ±35 kPa, confirming the measured mass of condensed CO_2_ and demonstrating an upper limit on any leak rate of approximately 0.0025 Pa·l s^−1^. The estimated liquid immersion at the triple point temperature with the cells fully melted is 17.3 cm.

### 2.2. SF_6_ cells

A single SF_6_ cell, identified as 803, was filled in 2019 for this study, based on the knowledge gained from our previous work [2]. The cell has a single 50 mm OD cylindrical volume that accommodates both liquid and vapor phases. A thermowell identical to the CO_2_ cells was installed, and 30 semicircular gold-plated copper baffles were installed inside the grooves to increase the heat exchange and provide additional support to the solid SF_6_ mantle at higher melted fractions (*F* > 50%). The measured tare mass of the empty cell was 2.31 kg.

Cell 803 was filled using the manifold described in [2] with gas from a 7 l source cylinder provided by Electronic Fluorocarbons, LLC. The gas certificate claims a minimum purity of 5N, with measured contents per volume of N_2_ of 1.6 μl l^−1^, 1.4 μl l^−1^ of H_2_O, and <0.5 μl l^−1^ for O_2_ and CF_4_. The claimed HF content was <0.1 μg g^−1^. We assign a nominal purity of 0.999 995 (5N5) on a molar basis to this sample.

The cell was cooled down to −47 °C and filled at a pressure of 0.26 MPa and a flow of 250 sccm for about 5.5 h. Once the cell was filled, the input valve was closed, and the cell was allowed to slowly warm up to room temperature. Cell 803 was weighed again and the total mass of SF_6_ condensed inside the cell was 573.3 g. Since some SF_6_ is still in liquid phase at room temperature, it was not possible to verify the amount of condensed gas using the cell manometer, as the internal pressure of the cell will be the SF_6_ vapor pressure at room temperature. The estimated liquid immersion at the triple point temperature with the cell fully melted is 20.8 cm.

## 3. Methods and procedures

### 3.1. ITS-90 traceability

The measurements were performed with one CSPRT, Fluke 5686 s/n 0103, installed in a precision-bore borosilicate tube immersion adapter identical to those of NIST SRM 1750 [17], and one LSPRT, Fluke 5681s/n 1528. CSPRT 0103 has a long calibration history, being used as one of the reference thermometers on [2], while LSPRT 1528 has a more limited history. Both thermometers were calibrated multiple times before and during the tests, at the triple points of argon (TP Ar, 83.8058 K), triple point of mercury, and at the melting point of gallium (MP Ga, 302.9146 K). Table 3 summarizes the calibration data for both SPRTs, where *a*_5_ and *b*_5_ correspond to the coefficients of the ITS-90 deviation function from the TP Hg to the MP Ga, and *a*_4_ and *b*_4_ correspond to the coefficients of the deviation function from the TP Ar to the TPW. Both SPRTs were calibrated using excitation currents of 0.707 mA and 1 mA, and their resistance measurements were extrapolated to 0 mA. The measurements were performed using an Isotech resistance bridge model microK Gold and a 100 ohms standard resistor Tinsley model 5685A temperature-controlled to within 10 mK in a MI model 9300A air bath.

**Table 3.** Calibration data of SPRTs 0103 and 1528.

	CSPRT 0103		LSPRT 1528	
	0.707 mA	0 mA	0.707 mA	0 mA
*W*_MP Ga_	1.118 118 679	1.118 118 702	1.118 130 213	1.118 130 446
*W*_TP Hg_	0.844 162 797	0.844 162 576	0.844 147 404	0.844 146 924
*W*_TP Ar_	0.215 968 786	0.215 967 980	0.215 893 570	0.215 889 447
*a*_5_	−1.545 945 043 × 10^−4^	−1.538 738 106 × 10^−4^	−5.645 371 960 × 10^−5^	−5.400 369 318 × 10^−5^
*b*_5_	−1.399 804 809 × 10^−4^	−1.444 583 471 × 10^−4^	−1.440 828 964 × 10^−4^	−1.481 108 283 × 10^−4^
*a*_4_	−1.319 989 261 × 10^−4^	−1.305 318 855 × 10^−4^	−3.286 344 692 × 10^−5^	−3.005 655 489 × 10^−5^
*b*_4_	4.612 550 010 × 10^−6^	4.898 811 187 × 10^−6^	6.696 521 251 × 10^−6^	5.097 188 600 × 10^−6^

CSPRT 0103 was installed into the cell thermowells, monitoring their temperatures most of the time, and all the measurements of full realizations used to determine the temperatures of the triple point of CO_2_ and SF_6_ were done employing an excitation current of 0.707 mA, which was also used for calibrating the SPRTs at the TP Ar, TP Hg and MP Ga. The measured self-heating coefficients ranged from 14 mK mW^−1^ to 28 mK mW^−1^ during the calibrations, resulting in a full-width amplitude in the observed self-heating of 0.12 mK, mostly due to the different external self-heating values measured when installing the SPRTs in different cells. LSPRT 1528 was directly installed into the bath fluid to monitor its temperature, ensuring that the fluid temperature was always a few millikelvin above or below the triple points for quasi-adiabatic realizations, and occasionally moved into the cells for calibrations at the TP CO_2_ and TP SF_6_.

### 3.2. Realization procedure

In the investigation reported in [2], the large immersion SF_6_ cells were realized using a method combining continuous melt and quasi-adiabatic modes for optimal measurements. In this work, a quasi-adiabatic pulsed realization approach was adopted. In this method, the cells were completely immersed in an ethanol bath to allow the fluid to flood the thermowells, preventing frosting inside the wells and improving the heat exchange between the cells and the SPRTs. The bath was maintained 3 mK–5 mK above the triple point realization temperatures to minimize the heat exchange between the cell and the bath fluid, and to provide a way of identifying when the plateau was over. The external jacket was not properly sealed to hold high vacuum, with some evidence of ethanol from the bath leaking inside, so instead the jacket was filled with ∼107 kPa of argon to insulate the cells from the bath fluid. This resulted in heat losses of the order of 20 % for CO_2_ and 60 % for SF_6_ when pulsing the cells using the heaters, so the calorimetry of the realizations is not accurate and is beyond the scope of this work.

For freezing the samples, two methods were adopted. The first approach consisted of setting the bath temperature to several kelvin below the triple point temperature to freeze the samples from the outside, while the second approach consisted of freezing the cell from the inside using solid CO_2_ (i.e. ‘dry ice’). It has been reported in [7] that the inner freeze method improves the realization of the TP CO_2_. For both CO_2_ and SF_6_, extra care must be taken when freezing the samples prior to the realization of their triple points. From the data provided by table 1, the CO_2_ contracts by 23 % upon freeze while SF_6_ contracts by 19 %, against only 3.5 % contraction exhibited by Hg.

**Figure 4.**
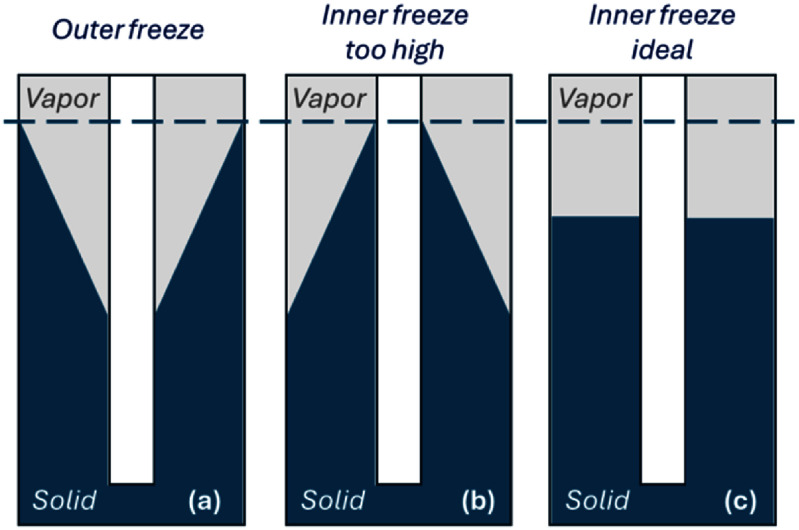
Impact of the freeze method on the shape of the solid mantle. Initial liquid-vapor line is show by the dashed line. Drawings are for illustration purposes only and are not to scale.

Figure 4 shows an idealized model that illustrates how the freezing method impacts the shape of the solid mantle. When freezing the cell from the outside, the liquid contracts as it freezes, so the height of the solid mantle reduces towards the thermowell. This results in a shorter solid–liquid interface, compromising the coupling between the SPRT and the phase transition and increasing potential immersion errors due to heat loss through the stem. When freezing the cell from the inside by simply pouring dry ice or liquid nitrogen (LN_2_) inside, there is a risk of freezing a mantle that is too tall and thin to support its own weight as it melts from the outside and would collapse in the middle of the realization (evidence of spontaneous disintegration of the mantle was shown in [2]), also compromising the immersion of the SPRT and increasing stem conduction errors. It would also not be straightforward to pour dry ice or LN_2_ inside the thermowell while the cell is completely immersed in the bath fluid.

The ideal scenario would be when the solid mantle is as cylindrical as possible, so the aspect ratio (width/height) of the mantle will be as large as possible, and spontaneous disintegration will be less likely. This is achieved by carefully controlling the height of the solid mantle as the sample is being frozen. This was accomplished by designing immersion coolers that would fit inside the thermowells, allowing the dry ice to be inserted in a controlled way. The immersion coolers consisted of a 7.8 mm OD closed-end brass tube for the TP CO_2_ cells, and a 7.5 mm OD closed-end glass tube for the TP SF_6_ cell (the brass tube would not fit inside the SF6 cell, possibly due to an uneven weld at the top of the thermowell). While the cell was kept 3 mK to 5 mK above the triple point temperature, fine-powdered dry ice was poured inside the immersion cooler placed inside the thermowell, and the height of the dry ice column was carefully controlled by means of marked glass rods sitting on the dry ice inside the coolers, as will be shown in the next section. This procedure was similar to the preparation of (TPW, 273.16 K) cell mantles, except that it was not possible to see the mantle as it grew, nor was it possible to accurately see how much dry ice was being poured. The maintenance of the baths above the triple point temperature was necessary to ensure there would be no outer freeze, and once the freeze was over the bath was set to ∼10 mK below the triple point temperature to ensure the cell remained frozen.

When following this method, the height of the ideal cylindrical solid mantle will be reduced from the initial liquid height by a factor equal to the ratio *ρ*_l,TP_*/ρ*_s,TP_. When comparing figures 4(a) and (b), given that the area of the cross sections of the solid mantles remains the same, the height of the solid mantle in direct contact the thermowell for an outer freeze is reduced from the initial liquid height by a factor of 2*ρ*_l,TP_*/ρ*_s,TP_. This will have an impact on the hydrostatic head corrections, as will be shown in the following sections.

Assuming an approximate value for the latent heat of sublimation of CO_2_ of 570 J g^−1^, it would be necessary to use approximately 23.7 g of dry ice to completely solidify the TP CO_2_ cells (for an estimated fusion enthalpy of 13.5 kJ), and approximately 36.6 g to solidify the TP SF_6_ cell (based on an estimated fusion enthalpy of 20.7 kJ). To compensate for heat leaks through the thermowell and the immersion cooler, as well for the cell heat capacity and the natural sublimation of the dry ice during the process, 50 g and 100 g of dry ice were used for freezing the samples, and the process took around 40 min and 1 h for the CO_2_ and SF_6_ cells, respectively.

**Figure 5.**
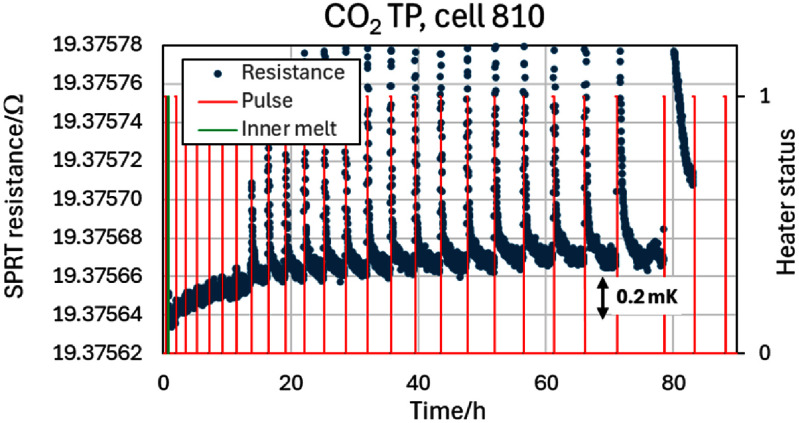
Pulsed realization of cell CO_2_ 810. Blue circles represent the SPRT readings, the red line represents the heater pulses, and the green line at the beginning of the realization represents the inner melt.

Once the cell was completely frozen and in equilibrium with the bath temperature, the bath was again set to 3 mK to 5 mK above the triple point, and an initial short pulse was applied to warm up the cell to a temperature even closer to the realization temperature. A sequence of pulses was then applied to completely melt the cells, with 1 W of power and variable durations, depending on the gas and the fraction to be melted per pulse. After the first pulse was applied, the SPRT was removed from the cell and an immersion heater was inserted to create an inner melt layer to improve SPRT immersion and minimize heat losses through the glass sheaths. The fractions melted on the inside ranged from 1 % to 2 % for TP CO_2_ cells and 3 % to 6 % for SF_6_ cells and were calculated from the estimated melting enthalpies of the cells and the power of the immersion heater. Figure 5 exhibits a pulsed realization of cell CO_2_ 810. The realizations with the CO_2_ and SF_6_ cells lasted at least about 100 h; since the bath temperature was controlled only a few millikelvin above the triple point temperature for approaching adiabatic conditions, the plateaus could be maintained for much longer. Both SPRTs used in this work were pre-cooled in the bath fluid before measurements in the cell to prevent excessive inner melt.

## 4. Results

### 4.1. TP CO_2_ results

**Figure 6.**
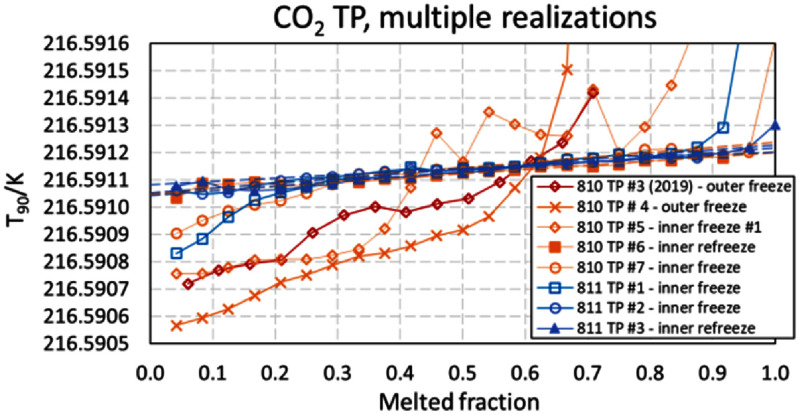
Realizations of the TP CO_2_ with cells 810 and 811. Data from 2025 except as indicated (2019). Solid symbols represent realizations at which the cell was frozen when the cell was not fully melted during the previous realization. Dashed lines correspond to linear extrapolations to *F* = 1.

Figure 6 summarizes realizations of the TP CO_2_ with cell 810 between 2019 and 2025, and with cell 811 in 2025. The realizations were initially planned to be over after 20 pulses of 675 J, equivalent to 5 % of the total cell enthalpy, with a couple of additional pulses to ensure the plateau was over. However, the observed number of pulses to fully melt both cells actually was 24, suggesting losses of 20 % of the applied power through the insulating argon gas, by conduction and/or convection. Cell 811 was further insulated with foam, wrapped around the surface where the heaters were installed, but no significant difference was observed. Because of the observed heat losses, the determination of the melted fractions *F* was based on the fraction of the total applied energy necessary to warm up the cell to 5 mK above the measured triple point temperature.

The first realizations with cell 810 were performed by freezing the cell from the outside. The observed supercooling ranged from 1.3 K to 3.3 K, depending on the bath set-point temperature. Once the cell was completely frozen, the bath was set to a temperature a few millikelvin above the triple point and heat pulses were applied to the cell to approximate the realization temperature before starting the pulse sequence. Once the plateau was observed, the check SPRT was removed and an inner melt was done with an immersion heater at 1 W for 135 s, which was equivalent to 1 % of the total cell enthalpy, and the check SPRT was moved back to the cell to measure the full realization plateau.

Two outer freeze realizations are shown as TP #3 (2019) and TP #4 in figure 6. It is possible to see that the melt ranges are much larger in comparison to the other realizations, being of the order of 1 mK. Such melt range seems usual for large immersion TP CO_2_ cells frozen from the outside in (see examples in [6, 7]). As it was illustrated in figure 4 and described in section 3.2, the shape of the solid mantle, when the sample is frozen from the outside, is such that the SPRT immersion is significantly reduced, especially for CO_2_, which contracts by 23.1 % in volume upon freeze at the triple point. Assuming that the shape of the frozen mantle follows exactly the ones illustrated in figure 4, i.e. discarding any interactions that would result in non-linear shapes, the immersion depth would be reduced by approximately 46 % at the beginning of the plateau. Additionally, as the CO_2_ is frozen from the outside, the diluted impurities are segregated towards the thermowell, meaning that the phase transition taking place closer to the SPRT will be impacted by a higher impurity concentration, decreasing its temperature at lower melted fractions.

To improve the quality of the plateaus, an inner freeze procedure was adopted, as proposed in section 3.2. A brass tubular immersion cooler was inserted in the cell and filled with fine-powdered dry ice. The first attempt of this method was done by keeping the height of the dry ice no lower than 17 cm, about the effective height of the liquid column when the sample is completely melted, and with no rigorously controlled upper limit. The result of the following realization is shown in figure 6 as TP #5. Although some improvement on the plateau could be observed, especially at higher melted fractions, it was still too unstable, showing a sudden temperature increase after pulse 8 and a drop after pulse 11, suggesting an irregular solid mantle and spontaneous disintegration as we reported in [2]. We attribute this plateau shape to the fact that the resulting mantle was too tall and thin, which caused it to collapse due to its own weight as it melted.

Accounting for the large contraction rate of CO_2_ (23.1 %, see table 1), an updated inner freeze procedure was adopted, where the height of the dry ice inside the immersion cooler was carefully controlled to provide a solid mantle that was as close as possible to a perfect cylinder. In this case, the height of the solid mantle would be reduced by approximately 4 cm, to reach 13.3 cm (note that for an outer freeze, the height of the mantle would be only 9.3 cm near the thermowell, see sections 3.2 and 5.1 for details). Therefore, the height of the dry ice was controlled between 11 cm and 14 cm in the following realizations using a marked glass rod to measure the height.

**Figure 7.**
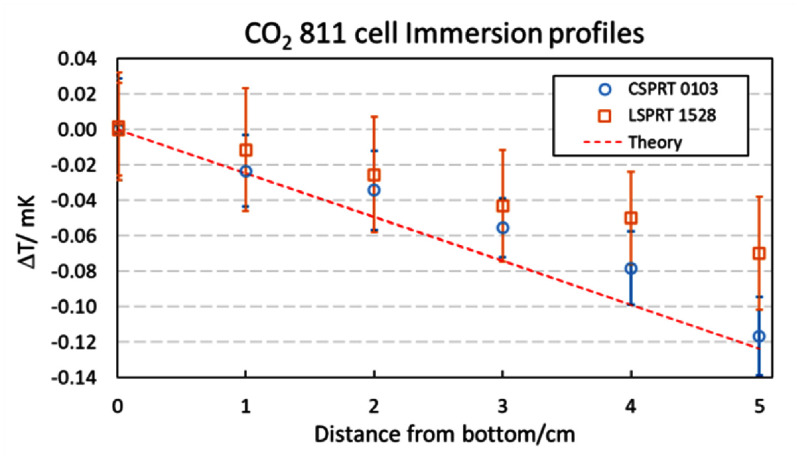
Immersion profiles of cell 811 with SPRTs 0103 and 1528. Measurements performed using an excitation current of 0.707 mA. Dashed line corresponds to the theoretical values based on the hydrostatic head correction for CO_2_ of 24.7 μK cm^−1^. Δ*T* values correspond to the temperature differences relative to the one measured with the SPRT at the bottom of the thermowell.

The results of the improved realization technique are also plotted in figure 6. After the controlled inner freeze was adopted, the reproducibility of the realization temperatures was better than 25 μK around pulse 12, at about 50 % of melted fraction, the agreement between the *F*= 1 extrapolations was better than 40 μK, and the average slope of the plateaus was estimated as 81 μK over 50 % of the melting range. It is very important to note that nearly 50 μK of this slope can be attributed to the expansion of the CO_2_ as it melts, which increases the hydrostatic pressure over the same 50 % of the melting range for which this slope was assessed. This means that the influence of impurities and other thermal effects are nearly negligible for these realizations in comparison to what we call a *progressive head correction*. We also have evidence suggesting that refreezing the CO_2_ sample before it was completely melted can further improve the slope of the plateau, especially at low melted fractions. This can be seen in figure 6 as solid symbols, and is in good agreement with the results reported in [4] using small adiabatic cells. The immersion profiles of both SPRTs are shown on figure 7, demonstrating that both thermometers track well the theoretical hydrostatic pressure line for the TP CO_2_, with deviations with relation to the expected value of 19 μK for CSPRT 0103 and 31 μK for LSPRT 1528 at 3 cm from the bottom of the thermowell, meaning that heat losses through the SPRT stems were negligible. The estimated *T*_90_ values for the TP CO_2_ are 216.591 19(15) K at *F* = 0.5 and 216.591 21(15) K at *F* = 1, for a coverage factor *k* = 1.

### 4.2. TP SF_6_ results

**Figure 8.**
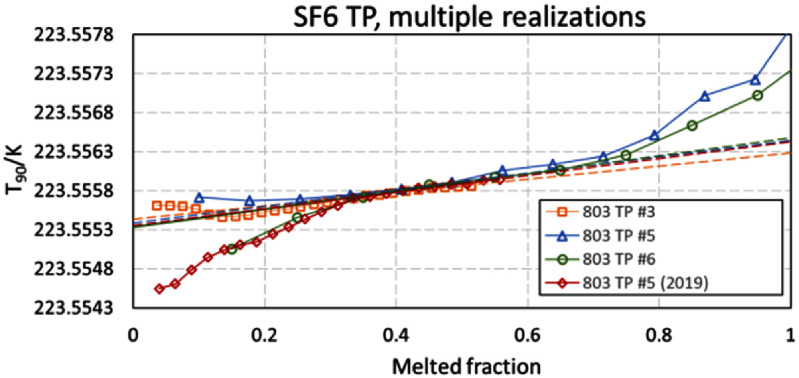
Realizations of the TP SF_6_ with cell 803. Data from 2025 except as indicated (2019). Dashed lines correspond to linear extrapolations to *F* = 1.

Figure 8 summarizes the results with cell SF_6_ 803. Similarly to what was done with the CO_2_ cells, initially it was estimated that the realization would be done with 20 pulses, each pulse equivalent to 1035 J, or 5 % of the total cell enthalpy, with a few additional pulses to ensure the sample was indeed melted. However, it was found that the heat leak was even larger than the ones observed with the CO_2_ cells. Since the OD of the liquid region of the SF_6_ cells was twice as large as the CO_2_ cells, the smaller gap between the cells and the external jacket favored even greater heat losses to the bath fluid through the insulating gas, being estimated as about 60 % of the power applied by the heaters. Because of this, part of the realizations was incomplete, but since all realizations reached at least 50 % of melted fraction, it was possible to extrapolate the data to estimate the temperatures at *F*= 1. The determination of the melted fractions was also based on the fraction of the total applied energy necessary to warm up the cell to 5 mK above the measured triple point temperature, once a full plateau was observed. Once the magnitude of the heat loss was properly estimated, full plateaus were realized to confirm the estimates given by the partial plateaus. The shape of the plateaus indicates that the copper baffles installed inside cell 803 successfully prevented the spontaneous mantle disintegration observed with large immersion SF_6_ cells, as reported in [2].

Considering the large contraction rate of SF_6_ (19 %, see table 1), although slightly smaller than that of CO_2_, and benefiting from the experience gained with the TP CO_2_ cells, a glass immersion cooler was employed to control the height of the mantle as the sample was frozen. In the case of SF_6_, a cylindrical solid mantle would have its height reduced also by approximately 4 cm, to reach 16.8 cm. Therefore, the height of the dry ice inside the immersion cooler was carefully controlled between 14 cm and 17 cm using a marked glass rod. Once it was verified that the cell was completely frozen with the check SPRT, the bath was set to 3 mK to 5 mK above the triple point, and heat pulses were applied to approximate the cell temperature before starting the pulse sequence. Once the phase transition started, the check SPRT was removed and an inner melt was performed with an immersion heater at 2 W for 500 s, which was equivalent to approximately 5 % of the total cell enthalpy, and then the check SPRT returned to the cell to monitor the plateau.

**Figure 9.**
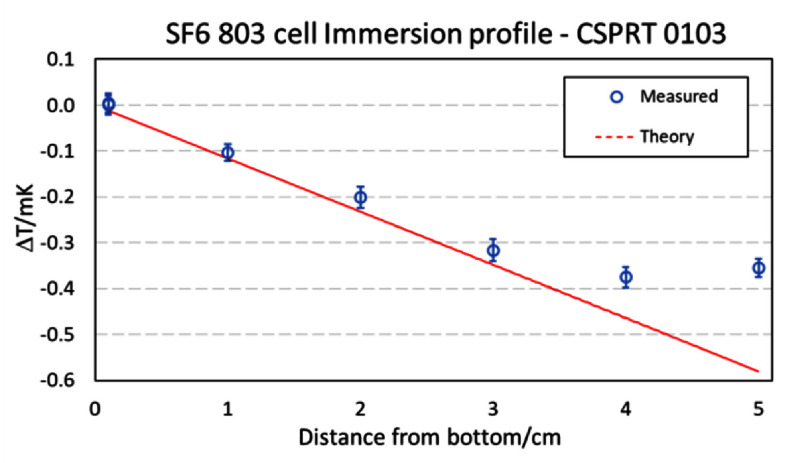
Immersion profile of cell 803 with CSPRT 0103. Measurements performed using an excitation current of 0.707 mA. Dashed line corresponds to the theoretical values based on the hydrostatic head correction for SF_6_ of 116 μK cm^−1^ [2]. ΔT values correspond to the temperature differences relative to the one measured with the SPRT at the bottom of the thermowell.

The reproducibility of cell SF_6_ 803 realizations in the central 50 % region of the melting range was better than 75 μK, and the agreement between the extrapolated *F*= 1 realization temperatures using the plateaus illustrated in figure 8 was better than 0.3 mK. The average slope of the plateaus was estimated as 0.46 mK over the central 50 % of the melting range, keeping in mind that the progressive head correction over 50 % of the melting range accounts for 0.23 mK of this slope, which is significant due to remarkably large d*T/*d*h* value of SF_6_ at the triple point [2, 18]. The immersion profile using the CSPRT 0103 is shown in figure 9, demonstrating that it also tracks well the hydrostatic pressure line, with a deviation with relation to the theoretical value of 32 μK at 3 cm from the bottom of the thermowell, not being significantly influenced by heat leaks. The estimated *T*_90_ value for the TP SF_6_ is 223.556 12(33) K for *F*= 0.5 and 223.556 22(33) K at *F* = 1, for a coverage factor *k* = 1.

### 4.3. ITS-90 traceability of SF_6_ and CO_2_ cells

During all realizations of the TP CO_2_ and TP SF_6_ reported in section 3, both SPRTs 0103 and 1528 were calibrated near 50 % of melted fraction, to investigate the feasibility of the use of these points for the calibration of SPRTs over subranges 4 (from the TP Ar to TPW) and 5 (from the TP Hg to MP Ga) of the ITS-90 [19]. To avoid circularity, we assign temperature values for the triple points of CO_2_ and SF_6_ from recently reported values for these fixed points in [2–10] and the values reported in this work. These assigned temperatures are 216.591 40(49) K and 223.556 00(65) K, respectively. Tables 4 and 5 summarize the temperatures as reported in the literature for the TP CO_2_ and TP SF_6_ together with those from this work. The nominal assigned temperatures were calculated as the simple average of the literature values, and the uncertainties were computed from a pooled variance, combined with the variance of the reported values.

**Table 4.** Reported literature and average values for the TP CO_2_. Uncertainties reported for a coverage factor *k* = 1.

Authors	Year	*T*_90_/K
Kawamura *et al* [4]	2019	216.590 90(36)
Okladnikov *et al* [5]	2022	216.591 70(30)
Liang *et al* [6]	2023	216.591 36(37)
Velcheva *et al* [7]	2024	216.591 70(35)
This work	2025	216.591 21(15)

Average:		216.591 40(49)

**Table 5.** Reported literature and average values for the TP SF_6_. Uncertainties reported for a coverage factor *k* = 1.

Authors	Year	*T*_90_/K
Rourke [8]	2016	223.555 23(49)
Tew and Quelhas [2]	2018	223.556 07(35)
Kawamura and Nakano [9]	2020	223.556 47(53)
Li *et al* [10]	2021	223.556 03(54)
This work	2025	223.556 22(33)

Average:		223.556 00(65)

The thermometers were measured multiple times at the melting point of Ga, and at the triple points of Hg, CO_2_, SF_6_ and Ar. Measurements at the TPW were performed after each measurement at the fixed points for accurate temperature determinations and to keep track of any possible drift during the assessment. All resistance measurements were performed using currents of 0.707 mA and 1 mA, which were then used to estimate the extrapolated resistances at 0 mA, and were corrected for hydrostatic head pressure.

**Figure 10.**
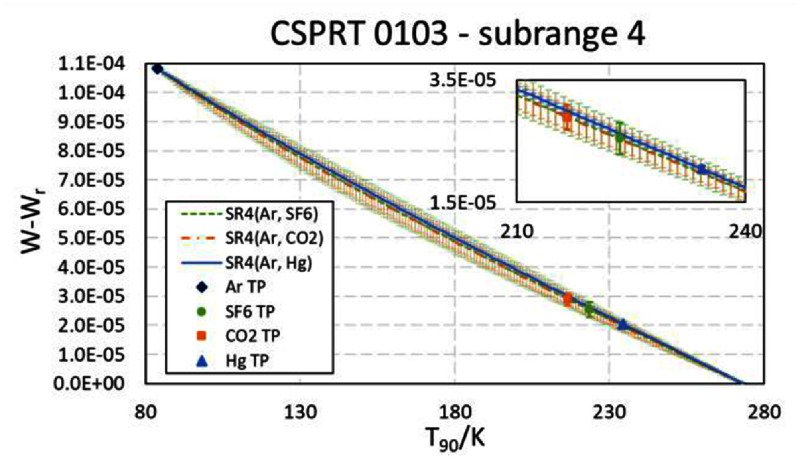
Results of the calibration of CSPRT 0103 in subrange 4 (TP Ar to TPW) using the TP Ar measurements with either the TP Hg, TP CO_2_ or TP SF_6_. Inset at the top right corner highlights the interpolated values in the region between 210 K and 240 K. Error bars correspond to the combined uncertainties (*k* = 1).

**Figure 11.**
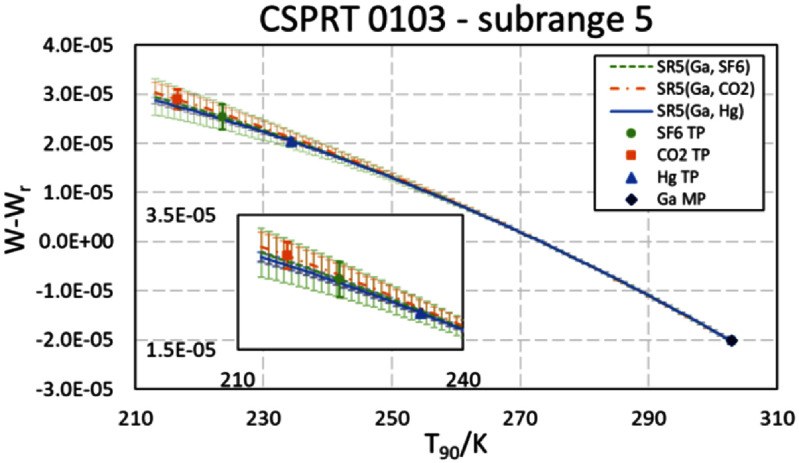
Results of the calibration of CSPRT 0103 in subrange 5 (TP Hg to MP Ga) using the MP Ga measurements with either the TP Hg, TP CO_2_ or TP SF_6_. Inset at the bottom left corner highlights the interpolated values in the region between 210 K and 240 K. Error bars correspond to the combined uncertainties (*k* = 1).

**Figure 12.**
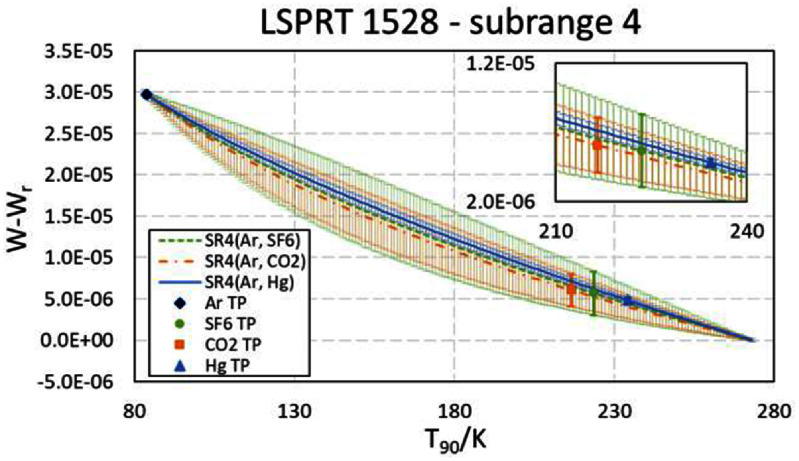
Results of the calibration of LSPRT 1528 in subrange 4 (TP Ar to TPW) using the TP Ar measurements with either the TP Hg, TP CO_2_ or TP SF_6_. Inset at the top right corner highlights the interpolated values in the region between 210 K and 240 K. Error bars correspond to the combined uncertainties (*k* = 1).

**Figure 13.**
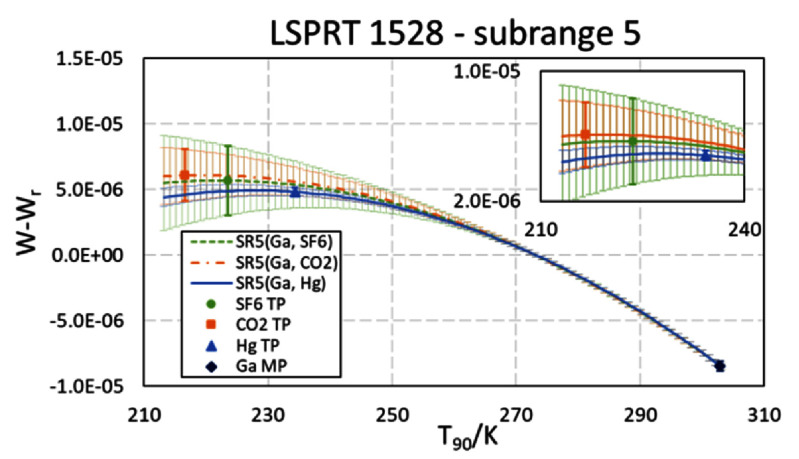
Results of the calibration of LSPRT 1528 in subrange 5 (TP Hg to MP Ga) using the MP Ga measurements with either the TP Hg, TP CO_2_ or TP SF_6_. Inset at the top right corner highlights the interpolated values in the region between 210 K and 240 K. Error bars correspond to the combined uncertainties (*k* = 1).

Figures 10–13 illustrate the results of the analysis. The measured resistance ratios *W = R*_FP_*/R*_TPW_, where *R*_FP_ is the resistance measured at the fixed point and *R*_TPW_ is the resistance measured at the TPW, were used to determine the coefficients of the ITS-90 deviation functions for subranges 4 and 5 using different combinations of fixed points. For subrange 4, the *W* values measured at the TP Ar were used with the values measured at either the TP Hg, TP CO_2_ and TP SF_6_ to calculate the coefficients *a*_4_ and *b*_4_ of the deviation function for each combination of fixed points. Analogously, for subrange 5, the values measured at the MP Ga were used with the values measured at either the TP Hg, TP CO_2_ and TP SF_6_ to calculate the coefficients *a*_5_ and *b*_5_ of the deviation function for each combination of fixed points. It is possible to see in the figures how the interpolated values using different combinations of fixed points agree with the measured values at these points. The differences between the measured values at the fixed points of Hg, CO_2_ and SF_6_ and the values interpolated using either of these fixed points were always smaller than the combined uncertainty of the differences, even when the fixed point measurement was not used to calculate the interpolated function. The degree of agreement between measured and interpolated values for both SPRTs was in general better on subrange 4 than on subrange 5, since the latter involved extrapolations from the TP Hg, which resulted in larger errors and uncertainties.

Tables 6 and 7 summarize the results of this analysis, providing the errors at each fixed point with relation to the interpolated functions, as well as the combined uncertainties (*k*= 1) of these errors, which account for contributions of both measured and interpolated values at the fixed point temperatures. The data in the tables show again that all errors are smaller than their estimated uncertainties, thus validating the use of both TP CO_2_ and TP SF_6_ for regular ITS-90 SPRT calibrations, provided that the realization temperatures are well known with reduced uncertainties, which this work demonstrated is possible.

**Table 6.** Error analysis for CSPRT 0103. Values correspond to the differences between the measured and interpolated values at the fixed points in mK. Combined uncertainties (*k* = 1) of the differences shown in parenthesis.

Subrange	Calibration points	CSPRT 0103		
		Hg	CO_2_	SF_6_
4	Ar, Hg	—	−0.26(50)	−0.20(66)
	Ar, CO_2_	0.19(37)	—	0.03(79)
	Ar, SF_6_	0.16(55)	−0.04(83)	—
5	Hg, Ga	—	0.34(51)	0.28(66)
	CO_2_, Ga	−0.19(28)	—	−0.02(74)
	SF_6_, Ga	−0.08(46)	0.43(98)	—

**Table 7.** Error analysis for CSPRT 1528. Values correspond to the differences between the measured and interpolated values at the fixed points in mK. Combined uncertainties (*k* = 1) of the differences shown in parenthesis.

Subrange	Calibration points	LSPRT 1528		
		Hg	CO_2_	SF_6_
4	Ar, Hg	—	−0.26(50)	−0.13(66)
	Ar, CO_2_	0.19(37)	—	0.10(79)
	Ar, SF_6_	0.11(54)	−0.12(82)	—
5	Hg, Ga	—	0.36(51)	0.20(66)
	CO_2_, Ga	−0.20(28)	—	−0.09(76)
	SF_6_, Ga	−0.13(45)	0.11(96)	—

## 5. Discussion

### 5.1. Impact of the progressive head correction

It has been postulated in sections 4.1 and 4.2 that the progressive head correction in immersion-type cells impacts the slope of the plateau by increasing the hydrostatic pressure as function of the melted fraction. This happens due to the spatial redistribution of the liquid mass as the solid melts leading to a *F*-dependence for a portion of the head correction. As a solid mantle melts, the height of the liquid column progressively increases, consequently increasing the hydrostatic pressure in the melting interface at the measurement point. This effect is normally negligibly small in metal fixed points, but because both CO_2_ and SF_6_ exhibit such relatively large volume expansions, (30 % and 23.5 %, respectively, see table 1) the effect is observable. For the TP CO_2_, the effect on the plateau slope is larger than any other effect, including the presence of impurities in the sample. For the TP SF_6_ the impact on the plateau is even greater in magnitude due to the particularly large value of d*T/*d*h*.

The correction is applied as a linear function of the melted fraction *F*, in contrast to a static value as traditionally done, by estimating the heights of the liquid columns relative to the center of the SPRT immediately after the beginning and at the end of the plateau. The progressive head correction can be estimated as

\begin{equation*}{{\Delta }}{T_{\mathrm{PHC}}}\left( F \right) = \left( {{h_i} + F \cdot \left( {{h_f} - {h_i}} \right)} \right) \cdot {\mathrm{d}}T/{\mathrm{d}}h{\text{ }}\end{equation*}

where *h*_i_ and *h*_f_ are the estimates of the effective heights of the initial (solid) and final (liquid) columns, respectively. The model assumes that: (i) there is a continuous inner liquid layer between the thermowell and the solid mantle, (ii) the height of the solid column surrounding this inner liquid layer remains constant over the entire plateau, and (iii) the solid mantle melts radially from the outer surface towards the thermowell.

For cylindrical solid mantles, as discussed in section 3.2 and illustrated in figure 4(c), an expression for the correction can also be given as

\begin{align*}{{\Delta }}{T_{\mathrm{PHC}}}\left( F \right) = \left( {{h_s} + F \cdot {h_s} \cdot \left( {\frac{{{v_l}}}{{{v_s}}} - 1} \right)} \right) \cdot {\mathrm{d}}T/{\mathrm{d}}h\end{align*}

where ${h_s} = {h_l} \cdot {v_s}/{v_l}$ is the effective height of an ideal solid mantle (figure 4(c)), *v*_s_ and *v*_l_ are the solid and liquid specific volumes, and (*v*_l_/*v*_s_*−*1) is the volume expansion on melting (7th column of table 1). If the cells were frozen using the outer freeze method, the shape of the solid mantle would be similar to figure 4(a), and the estimated difference between the initial and final heights of the liquid column would be twice as large (see section 3.2), so as the impact on the plateau slopes, and equation (2) would not apply directly.

The progressive head correction is applied to measurements similarly to the static head correction, which is traditionally calculated based on estimates of the height of the completely liquid sample surrounding the thermowell at the fixed point temperature. It is ideally applied for a melted fraction *F* ranging from 0^+^ to 1^−^ (i.e. immediately after the onset of the melt to immediately before the end of the melt). In practice, however, this would apply for a melted fraction *F* ranging from few % to the point where immersion begins to deteriorate. In the limit *F* = 1, the progressive head correction reduces to the static head correction.

**Figure 14.**
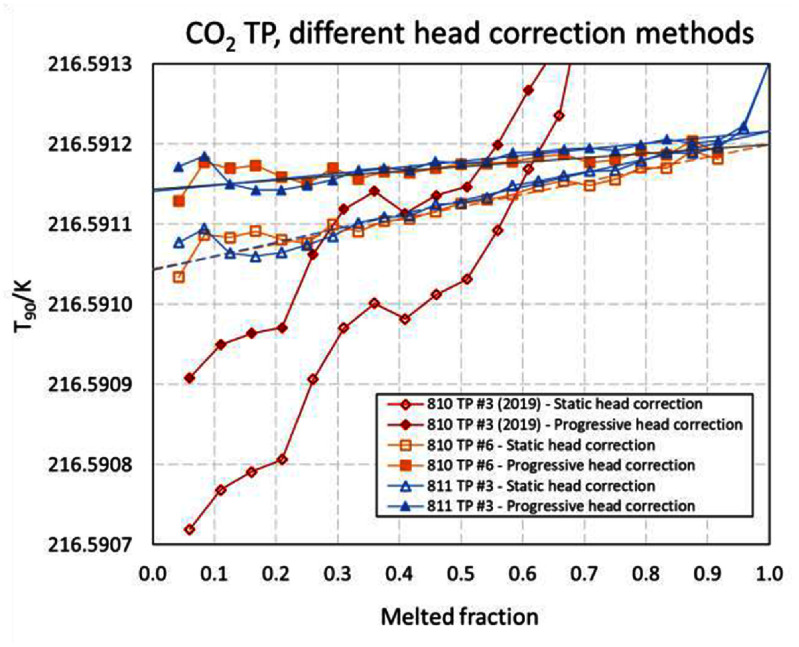
Effect of the progressive head correction on three measurements of the triple point of CO_2_. Open symbols and dashed lines correspond to the measured temperatures after applying the normal head correction. Solid symbols and solid lines correspond to the measured temperatures after applying the progressive head correction. The realization ‘810 TP #3 (2019)’ was done using the outer freeze method, while the other two were done using the inner freeze method.

**Figure 15.**
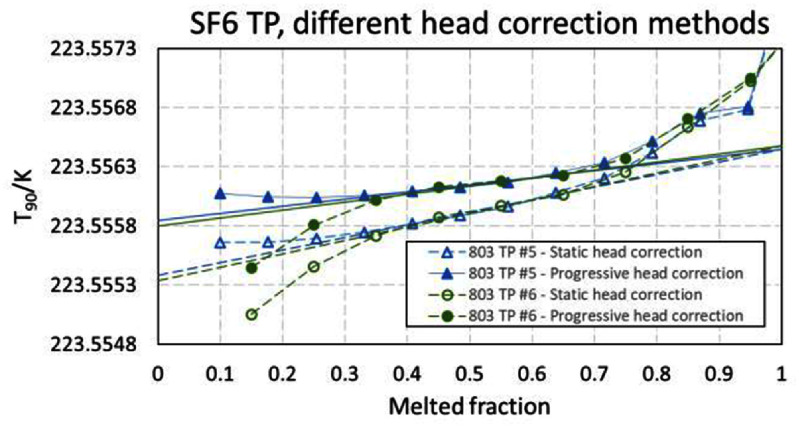
Effect of the progressive head correction on two measurements of the triple point of SF_6_. Open symbols and dashed lines correspond to the measured temperatures after applying the normal head correction. Solid symbols and solid lines correspond to the measured temperatures after applying the progressive head correction.

Figures 14 and 15 exhibit the impact of the progressive head correction on the slopes of plateaus of the triple points of CO_2_ and SF_6_, respectively. It can be seen that the effect is more pronounced at low melted fractions, and the temperatures at higher melted fractions converge to the same values at *F*= 1. It is important to note that applying this correction did not change the extrapolated temperature values for the triple points of CO_2_ and SF_6_ at *F* = 1, only the slope of the plateau is changed. The most practical impact of this correction would be on regular calibrations of SPRTs, since the measurements take place at lower melted fractions, usually between 30 % and 70 %. By applying the progressive head correction, the measured temperatures would be closer to the liquidous point temperatures.

Figure 14 also includes one realization done using the outer freeze method (marked as ‘810 TP #3 (2019)’). Since the shape of the solid mantle is different for this realization method (see figure 4(a)), the difference between the estimated initial and final heights of the liquid column was twice that of the other two realizations using the inner freeze method (see section 3.2). In applying the correction as estimated the inflection point of the realization curve moved much closer to the other two—realized using the inner freeze method, which further supports our observations that the magnitude of this effect is strongly dependent on the freeze method. A similar approach could not be applied for the SF_6_ realizations using the outer freeze method with cell 803 because the presence of the copper baffles inside the cell likely distorts the solid mantle shape in a way that lacks correspondence to figure 4(a), being most likely approximately cylindrical. The presence of the baffles should not influence significantly the shape of the solid mantle obtained using the inner freeze method, as the height of the dry ice column is carefully controlled.

For the TP CO_2_ cells studied in this work, the estimated initial height when employing the inner freeze method was 13.3 cm at *F*= 0 and the final height was estimated as 17.3 cm at *F* = 1, resulting in a slope of 0.1 mK. For the TP SF_6_, the estimated initial and final heights were 16.8 cm and 20.8 cm, respectively, with a slope of 0.46 mK. These values were estimated for cylindrical solid mantles, and were obtained by the controlled inner freeze method using immersion coolers filled with dry ice. For comparison purposes, from the values for the inverse of the first cryoscopic constant from table 1, the estimated plateau slope based on Raoult’s Law [20] for a 99.9999% (6 N) pure sample of CO_2_ would be 45 μK, and the slope for a 99.9995% (5N5) pure sample of SF_6_ would be 0.4 mK.

### 5.2. Impurity distribution effects

The dominant chemical impurity in all three of the TP cells studied here is nitrogen, N_2_. Direct cell-sampled assays are not available, so this is inferred on the basis of the chemical assays of the source gases and the known efficacy of the gas purifiers we have utilized. The liquid-vapor distribution coefficients *K* for both SF_6_ and CO_2_ in the dilute limit are known well enough to estimate values of *K*(*T*) at the TP temperatures. The solid-phase solubilities are unknown and the results from our analysis of the plateau *F* dependence as will be shown in section 5.5 are ambiguous. For the purposes of uncertainty estimation, we assume N_2_ to be insoluble in the solid. Under this assumption, the partitioning of a volatile impurity such as N_2_ produces two effects on the observed melting temperature. First, the fraction of the impurity that is dissolved in the liquid depresses the melting point temperature and second, the presence of the impurity in the vapor gives rise to a partial pressure *p*_N2_ that will slightly elevate the melting point given the positive values of d*T*/d*p* for both solutes.

For N_2_ in both SF_6_ and CO_2_, *K*(300 K) ∼ 4, so *a* ∼ 4 times higher mole fraction of the impurity in vapor phase is present at equilibrium near ambient conditions. The impurity analysis takes this into account and the assay is accomplished by sampling the liquid phase where the impurity level should be closer to that of the bulk source material [21,22, 23]. Nonetheless, some degree of ambiguity in the actual transferred impurity content will always occur when filling a volume via gas-phase flow from a source cylinder having both liquid and gas phases. When drawing from the vapor phase of the source cylinder, a higher impurity mole fraction may be initially transferred. Depending on the degree of departure from equilibrium as the vapor is withdrawn, impurity levels slightly lower than the bulk value could also be transferred [2].

In the case of SF_6_, our previous study [2] gave estimates of *K*_N2,SF6_(223.5 K) = 35 and *K*_N2,SF6_(300 K) = 4. The assay for our contemporary SF_6_ source gas used to fill cell 803 was *X*_N2_ = 1.6 μmol mol^−1^, as reported in section 2.2, which is a factor of 0.6 lower than that of the SF_6_ source used in [2]. Assuming that this same impurity level is transferred into the cell, the dissolved N_2_ in the liquid phase at 223.556 K would be *X*_N2L_ = 1.3 μmol mol^−1^ with the remaining fraction of N_2_ in the vapor phase at *X*_N2V_ = 44 μmol mol^−1^ or *p*_N2_ = 11 Pa. That would lead to a melting point depression of 0.1 mK in the Raoult limit but only 0.007 mK of elevation due to the partial pressure effect.

In the case of N_2_ impurities in CO_2_, Martynov *et al* [24] have complied data for the Henry’s Law constants for a variety of volatile impurities including N_2_. From that data it is possible to derive values for the distribution coefficients which we estimate are *K*_N2,CO2_(216.59 K) = 127 at the triple point temperature, and *K*_N2,CO2_(295 K) = 3.7 for ambient temperature. The assay for our initial CO_2_ source gas used to fill cell 810 was *X*_N2_ = 1.1 μmol mol^−1^, and *X*_Ar_ = 0.3 μmol mol^−1^, as reported in section 2.1. But most of these volatiles would have been removed in our degassing procedure at 87 K, leaving behind ∼1/40 of the original volatile impurities. The assay for the higher purity gas used in our more contemporary cell, 811, was *X*_N2_ = 0.03 μmol mol^−1^, and this is the N_2_ impurity fraction that we assume for both of our CO_2_ cells. At 216.592 K this would result in dissolved N_2_ in the liquid phase of *X*_N2L_ ≈ 0.004 μmol mol^−1^ with the remaining fraction of N_2_ in the vapor phase at *X*_N2V_ = 0.47 μmol mol^−1^ or *p*_N2_ = 0.27 Pa. These small concentrations would produce negligibly small temperature depressions/elevations (i.e. <1 μK) in our two CO_2_ cells.

### 5.3. Isotopic variability effects

No isotopic assays are available for any of the source gases used in this work. There are, however, several relevant reports in the literature that may be used to set nominal bounds on the degree of the expected isotopic variability in these and similar commercial gas sources and chemical reagents [25, 26]. Variations are reported as relative differences *δ* in isotope ratios with respect to an isotope reference material having a known isotopic content expressed as a ratio or as a mole fraction *X*_ref_. The isotopic variations may, in principle, lead to variability in the realization temperatures. In this section we provide estimates of the isotopic effects on the realization temperatures that indicate these effects are negligibly small.

In the case of CO_2_, the two most relevant isotopic substitutions are ^13^C/^12^C and ^18^O/^16^O, forming the two most significant of the minor isotopologues C^18^O^16^O and ^13^CO_2_., with abundances of approximately 0.2 % and 1 % respectively. The relative differences *δ*^13^C are usually reported relative to the isotopic reference limestone known as Vienna Peedee belemnite (VPDB) and the *δ*^18^O values are reported relative to Vienna Standard Mean Ocean Water (VSMOW). The two most relevant CO_2_ isotopic reference gases are RM 8663 and RM 8564 [27], the former being derived from petroleum and the latter being derived from C_4_ biomass. For commercial CO_2_ cylinders, Socki and Jacksier [28] have shown that gas taken from three separate cylinders under a wide variation of temperature and pressure conditions will always fall within a narrow band of 4.2‰ ⩽ *δ*^18^O_VSMOW_ ⩽ 5.4‰ and −41.55 ‰ ⩽ *δ*^13^C_VPDB_ ⩽ −41.13 ‰. These results demonstrate that any isotopic fractionation effects in transferring CO_2_ from transport cylinders are small. These three gas cylinders exhibited average values of *δ*^13^C and *δ*^18^O close to that of RM 8563, implying a petroleum origin to their gas source.

The known variability in the isotopic composition of commercial CO_2_ compressed gas/liquid has been summarized by the IUPAC [26], where they state the observed maxima and minima in the isotopic content as 3.9 ‰ ⩽ *δ*^18^O_VSMOW_ ⩽ 22.2 ‰ and −54 ‰ ⩽ *δ*^13^C_VPDB_ ⩽ −29 ‰. These variabilities are factors of ×9.4 and ×6.8 smaller than those estimated in Liang *et al* [29] due to their estimates being based on the entire range of variations exhibited amongst all terrestrial sources of oxygen and carbon. The more recent reports of CO_2_ cylinders from NIM [6] and NMIJ [4] indicate that some high-purity gas sources are significantly more depleted in the heavier isotopes than the IUPAC reports have indicated. By combining all of the published isotopic data to date, we infer an expanded range of isotopic variability as, −30 ‰ ⩽ *δ*^18^O_VSMOW_ ⩽ 31.1 ‰ and −54 ‰ ⩽ δ^13^C_VPDB_ ⩽ −3.8 ‰. We note that these nominal ranges are non-overlapping with the heavier CO_2_ found in the earth’s atmosphere, where *δ*^18^O_VSMOW_ ∼ +41 ‰ to +53 ‰ [25], which is as expected since commercial CO_2_ is not extracted from the air.

In the case of SF_6_, ^32^S makes up about 95 % abundance in terrestrial sources and there is only one significant minor isotopologue, ^34^SF_6_, normally with about 4.2 % abundance. But compared to CO_2_ there are much less data on the degree of isotopic variability in commercial SF_6_ gas cylinders. Ratios of ^34^S/^32^S are usually reported as *δ*^34^S_VCDT_ relative to the Vienna Canyon Diablo Troilite (VCDT) reference material [25]. Eiler *et al* [30] reported on three high-purity SF_6_ gas samples of unspecified origin that were used for their study of isotope fractionation in SF_6_. Their SF_6_ gas samples exhibited variations of 1.6 ‰ ⩽ *δ*^34^S_VCDT_ ⩽ 17 ‰. All other reports on isotopic compositions of SF_6_ gas appear to come from gases produced in the laboratory from either sulfur-bearing compounds or inorganic reference materials such as AgS_2_. Commercial SF_6_ is synthesized by fluorination of reagent grade elemental sulfur which exhibits a range of isotopic variation of −8 ‰ ⩽ *δ*^34^S_VCDT_ ⩽ 28 ‰ [26]. We assume that most commercial SF_6_ exhibits *δ*^34^S_VCDT_ values that fall in this range. These variabilities are factors of ×7.6 smaller than those estimated in Liang *et al* [29] due to their estimates being based on the entire range of variations exhibited amongst all terrestrial sources of sulfur.

The isotopic variations in the molecular isotopologues will produce triple point temperature shifts Δ*T* as discussed in White and Tew [31] and in Liang *et al* [29] according to

\begin{equation*}\Delta T = \left( {RT_{{\mathrm{TP}}}^2/\Delta {h_{\mathrm{f}}}} \right)\ln \left( {{\alpha _{{\mathrm{s}} - {\mathrm{l}}}}} \right){X_{{\mathrm{ref}}}}\delta \equiv A\delta ,\end{equation*}

where *α*_s–l_ is the solid–liquid separation factor, *A* is the isotopic correction constant, and *R* is the molar gas constant. As discussed in Liang *et al*, there are no data on *α*_s*–*l_ for either CO_2_ or SF_6_ so the values must be inferred indirectly by combining solid–vapor (s–v) and liquid–vapor (l–v) isotopic separation data for the special case (applicable only at the triple point temperature) where ln(*α*_s*–*v_)−ln(*α*_l*–*v_) = ln(*α*_s*–*l_). The problem then becomes identifying literature data sources for ln(*α*_s*–*v_) and ln(*α*_l*–*v_), each having unique temperature dependencies, which are sufficiently close to the TP temperatures and numerically coherent. Ideally, this means that the both liquid–vapor and solid–vapor data should be taken from the same experimental work.

In the case of CO_2_, the work of Bilkadi *et al* [32] (BLB75) provides the most complete set of liquid and solid-phase isotope separation data, having made measurements from 154 K to 217 K going through the TP temperature. BLB75 also made detailed theoretical calculations, and their experimental results were in good agreement with the theoretical predictions. They found a normal isotope effect for C^18^O^16^O and a reverse effect for ^13^CO_2_ over the full range of temperatures studied. Using their reported fit parameters we have calculated the separation factors at their reported value for the TP CO_2_ temperature of 216.63 K. The results are tabulated in table 8. These values for ln(*α*_s–l_) are used in equation (3) to calculate values of *A*_18OCO_ = 93 μK and *A*_O13CO_ = −23 μK which are factors of ×2.4 and ×8.4 smaller, and opposite in sign, than those estimated in Liang *et al* [29]. These differences in the *A* constants result from the use of different data sources and different assumptions made for calculating the separation factors. The proportionately larger influence of ^18^O substitution compared to that of ^13^C is expected due to the exterior positioning of the oxygen atoms that govern intermolecular forces. Combining our values of *A*_18OCO_ and *A*_O13CO_ with our expanded range for the variations of *δ*^18^O_VSMOW_ and *δ*^13^C_VPDB_ in commercial CO_2_ via equation (3) yield estimates for the isotopic variability in the TP temperature in the ranges −2.8 μK < Δ*T*_18O_ < +2.9 μK and ∼1.3 μK < Δ*T*_13C_ < 0.1 μK. These values should be as accurate and reliable as is the source data from BLB75 [32], the uncertainty from which remains unassessed, but would be expected to be ∼<10 % on the basis of their C^18^O_2_ data near the triple point.

**Table 8.** CO_2_ Isotope separation factors taken from BLB75 [32].

Isotopologues	Separation factors at TP CO_2_		
	*α*_l–v_	*α*_s–v_	*α*_s–v_
C^18^O^16^O	1.001 63	1.002 68	1.001 05
^13^CO_2_	0.999 67	0.999 62	0.999 95

There are much less isotopic separation data available on SF_6_ and those data sources are both conflicting in sign and magnitude and inconsistent with theory [30, 33, 34]. Given the symmetric molecular structure and the dominant role of the monoisotopic fluorine atoms in bonding, however, it is expected that any fractionation effects in SF_6_ should be exceptionally small.

The most complete set of SF_6_ isotopic separation data are from Eiler *et al* [30], who reported liquid-phase data at 300 K and 228 K and solid-phase data that were mostly taken at 155 K. They reported ^34^S/^32^S values of ln(^34^*α*_l–v_) = 1.1 × 10^−4^ at 228 K between the liquid and vapor phases, which they describe as ‘negligibly small’. When their liquid phase data for ln(^34^*α*_l–v_) are interpolated via *a*/*T*^2^ + *b*/*T* (where *a*= 57.5 K^2^ and *b*= −0.226 K), we obtain ln(^34^*α*_l–v_) = 1.4 × 10^−4^ at 223.556 K. In contrast to this, the solid phase data from Eiler *et al* are not readily reconcilable with well-established theories for the temperature dependence. We are unable to find a suitable method to extrapolate their data from 155 K to 223.556 K. Using SF_6_ ‘ice’ data in the range 155 K and 137 K, or with additional data from absorbates near 180 K, physically acceptable interpolations/extrapolations of the form *a*/*T*^2^ + *b*/*T +c*, where both *a*> 0 and *c* are reasonably small, were not found.

Given that there is no reliable way to utilize the solid-phase separation data from Eiler *et al* [30], we resort to a simplified approximation due to Jeevanandam [35] that relates the two condensed phase separation factors via ln(*α*_s–v_)≈(*v*_l_*/v*_s_)ln(*α*_l–v_), which is only valid at the triple point temperature. This then leads to our estimate for the solid–liquid separation factor ln(^34^*α*_s–l_) ≈ 3.3 × 10^−5^, which is similar in magnitude to that of ^13^C in CO_2_, as expected for atoms having an interior position within their respective molecules. This approximation works well for the monoatomic rare gases and in a few cases for simple molecules with weak liquid-phase association [35]. We note that the Jeevanandam approximation does not work well for CO_2_, where it overestimates ln(^13^*α*_s–l_) by ∼×2 for ^13^CO_2_ substitution but underestimates ln(^18^*α*_s–l_) by ∼×2 for C^18^O^16^O substitution, when compared to the results derived from BLB75. Given this known limitation and the structure of SF_6_, the actual value for ln(^34^*α*_s–v_) could differ by factors of ∼±2 compared to this approximation.

Given assumptions we again use equation (3) to calculate *A*_34S_ = 108 μK and *A*_33S_ = 10 μK for the SF_6_ isotopic correction constants. These estimates, when combined with the known variation *δ*^34^S_VCDT_ of commercial reagent sulfur, predict isotopic variations for the triple point temperature of commercial SF_6_ gas to be within the range −6 μK < Δ*T*_34S_ < −3 μK, and much less than this for Δ*T*_33S_. These estimates are a factor of ∼×546 smaller than those made by Liang *et al* [29]. This is due to our estimated value of ln(^34^*α*_s–l_) being a factor ∼×(−72) smaller and the range of δ^34^S_VCDT_ being ∼×7.6 smaller, as stated above. The factor of −72 is due to different assumptions in utilizing the data from Eiler *et al* [30] and to our use of the Jeevanandam approximation. Given all of the uncertainties and approximations involved, our estimates for these SF_6_ isotopic variations should be considered valid only to within factors of ∼3 (i.e. an ‘order-of-magnitude’ estimate).

### 5.4. Calorimetry

We have employed calorimetric methods to estimate the melted fractions *F* to specify the realization conditions over the melting plateaus. The nominal *F* values are calculated as *F*= Δ*Q*/(*m*_cell_Δ*h*_f_) via the applied heating pulse energies Δ*Q* and the cell enthalpy of melting *m*_cell_Δ*h*_f._ The *F* values are uncertain at the level of ∼5 % due to the uncertainty in the heat shunting effect in our cell heaters (see sections 4.1 and 4.2), limiting our ability in determining the effective heat Δ*Q*_eff_ absorbed by the cell and transferred to the solid mantle. In practice we apply corrections to determine Δ*Q*_eff_ = Δ*Q*_nom_(1−*f*_shunt_), where Δ*Q*_nom_ is *P*Δ*t*, the power *P* and dwell time Δ*t* applied to the heaters, and *f*_shunt_ (=0.6 for cell 803 and 0.2 for cell 810/811) is the fraction of applied heat lost to the bath through the shunting effect. Since *f*_shunt_ is determined empirically through trial and error, it is the principal source of the uncertainty in *F*.

The values of the specific enthalpy of fusion Δ*h*_f_ are also uncertain at the 1 % to 2 % level. In the case of CO_2_, the Δ*h*_f_ = 200 J g^−1^ from table 1 is chosen to be consistent with the recently revised CO_2_ melting line from Harvey [14], but is also consistent with the value as measured by Ancsin [36].

The calorimetry needed to affect the realizations is thus inherently inaccurate, but this does not pose any significant limitation to the overall uncertainty in temperature. These effects are accounted for in the uncertainty budget under the component labeled ‘*Extrapolation to F* = 1’, see section 5.6.

### 5.5. Determination of the triple point temperatures

The estimated temperature values for the triple points of CO_2_ and SF_6_ in this work resulted from extrapolations of the measured temperatures at different melted fractions to the liquidus point at *F*= 1. For these extrapolations, three methods were considered, following the recommendations of [20]. The first and simplest method consisted in a linear extrapolation of the observed temperatures *T*_obs_ as function of *F*. This method is often used for analyzing melting lines of samples that are known to be very pure or less sensitive to impurities. The second method was a linear extrapolation of *T*_obs_ as function of the reciprocal melted fraction *F*^−1^. The method is traditionally adopted in the literature and assumes that all impurities are insoluble in the solid phase, so their molar fraction in the liquid phase reduces as the melting proceeds. The third method would better model cases where the impurities are soluble in the solid phase, so the solid/liquid distribution coefficient *k_s,l_* is a finite number between zero and one. The extrapolation is done linearly as function of *F^−y^*, where −*y*= (*k*_s.l_*–*1). In our previous work, we found that adopting *y* = 0.8 yielded lower statistical uncertainties in comparison to *F*^−1^ fits for three out of the four SF_6_ cells that were investigated [2], so those fits were included as well in the analysis.

Table 9 shows the values for the estimated *F*= 1 temperatures for all triple point cells studied in this work using the three models. The table includes the values obtained without accounting for the progressive head correction and the values found after applying the correction. It was already demonstrated in 5.1 that the application of the progressive head correction does not affect the linear *F* = 1 extrapolations, but this was not the case for the extrapolations as function of the reciprocal melted fraction *F^−y^* (0 < *y* ⩽ 1). For consistency, the same regions of the plateaus were used with all models to provide estimates of the liquidus points for all cells. For the TP CO_2_, the data between 0.3 < *F* < 0.8 was used, while for the TP SF_6_, the interval adopted was 0.35 < *F* < 0.65. The choice of such a limited range for the TP SF_6_ was based on the fact that the plateaus were approximately linear over this melted fraction interval.

**Table 9.** Temperature estimates for the triple points of CO_2_ and SF_6_. Values in parenthesis correspond to the statistical uncertainties of the fits. *F*= 1 fits not affected by the progressive head (PH) correction. *F^−y^* values calculated for *y* = 0.8. Bold values are the values adopted as estimates for the fixed point temperatures.

	Fitting model				
		*F^−^*^1^ *=* 1		*F^−y^ =* 1	
Cell	*F =* 1	No PH correction	PH correction	No PH correction	PH correction
CO_2_ 810	216.591 218(21)	216.591 187(10)	216.591 205(10)	216.591 190(13)	216.591 206(13)
CO_2_ 811	216.591 215(10)	216.591 187(4)	216.591 205(4)	216.591 189(5)	216.591 206(5)
CO_2_ average	216.591 217(10)	216.591 187(5)	**216.591 205(5)**	216.591 190(6)	216.591 206(6)

SF_6_ 803	223.556 445(52)	223.556 068(48)	**223.556 217(35)**	223.556 091(59)	223.556 201(46)

**Figure 16.**
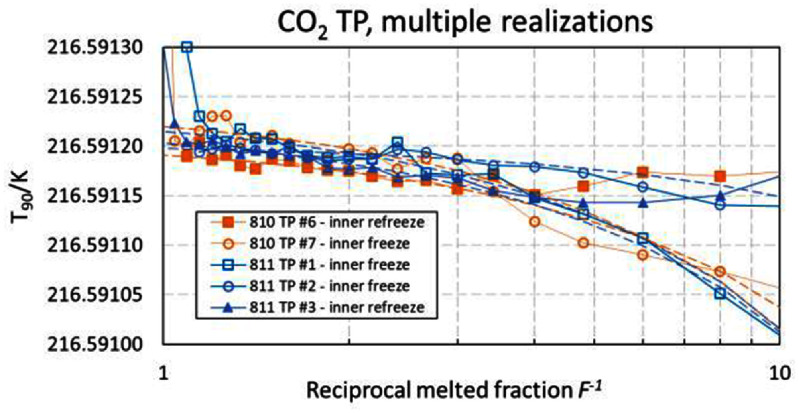
*F*^−1^ analysis for the TP CO_2_. Dashed lines represent the linear *F*^−1^ fits extrapolated to one for determining the liquidus point of the CO_2_ samples.

**Figure 17.**
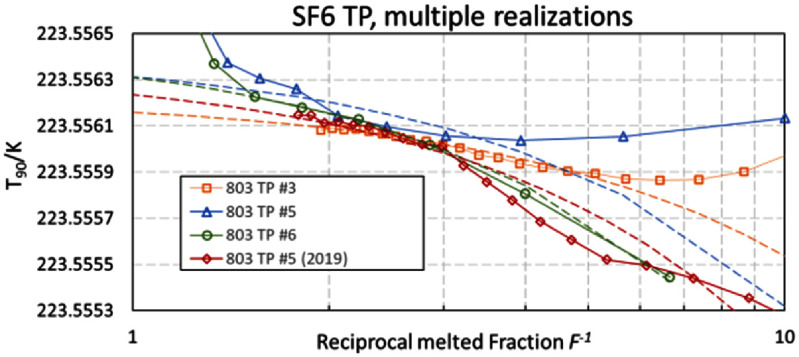
*F*^−1^ analysis for the TP SF_6_. Dashed lines represent the linear *F*^−1^ fits extrapolated to one for determining the liquidus point of the SF_6_ sample.

Figures 16 and 17 illustrate the fits adopted for determining the temperatures of the TP CO_2_ and TP SF_6_, respectively. The chosen models were the ones that provided the lower statistical uncertainties, as recommended by [20]. For both fixed points, the best fits were obtained with the *F*^−1^ model after applying the progressive head correction. Attempts to fit the data using *y <*1 showed a consistent increase in the statistical uncertainties with the decrease of *y*. Regardless of the model adopted, all temperature estimates for the TP CO_2_ agreed to less than 30 μK, while the agreement between the TP SF_6_ estimates was of the order of 0.15 mK. Moreover, the agreement between the TP CO_2_ estimates for the cells 810 and 811 was of the order of 3 μK or better. Based on this analysis, the values adopted for the TP CO_2_ were 216.591 19(15) K for *F*= 0.5 and 216.591 21(15) K for *F* = 1, and for the TP SF_6_ the estimated realization temperatures were 223.556 12(33) K for *F*= 0.5 and 223.556 22(33) K for *F* = 1.

### 5.6. Uncertainties

Table 10 details the uncertainties of the realizations of the triple points of CO_2_ and SF_6_. For both fixed points, the largest contributions come from the ITS-90 interpolation uncertainties, from the calibrations of the CSPRT 0103 at the TP Hg and TP Ar, and from the slopes of the plateaus, especially for the TP SF_6_.

**Table 10.** Uncertainties of the TP CO_2_ and TP SF6 realizations. Values in millikelvin.

Component	TP CO_2_	TP SF_6_
Bridge repeatability	0.004	0.004
Bridge non-linearity	0.019	0.019
Standard resistor	0.006	0.006
Phase transition repeatability	0.036	0.074
Chemical impurities	0.001	0.059
Gas pressure	0.001	0.004
Isotopic variation	0.004	0.016
Hydrostatic head correction	0.014	0.067
Self-heating correction	0.033	0.033
Immersion error	0.015	0.073
Slope of the plateau	0.047	0.266
Extrapolation to *F* = 1	0.005	0.036
ITS-90 realization	0.103	0.093
SPRT non-uniqueness	0.078	0.055
TPW propagation	0.031	0.032

Combined uncertainty (*k* = 1)	0.15	0.33

From the uncertainty budget, it is possible to determine that the combined uncertainties of the triple points of CO_2_ and SF_6_ can be as low as 0.07 mK and 0.20 mK, respectively, provided that there are consensus values for these fixed points in the ITS-90 that would eliminate the need of interpolating from measurements at other points, and that the progressive head correction is properly accounted for in the measurements, reducing then the uncertainty contribution due to the slope of the plateau to 0.018 mK and 0.130 mK for the TP CO_2_ and TP SF_6_, respectively. A detailed description of the uncertainty components is presented as follows:

#### 5.6.1. Bridge repeatability

**Figure 18.**
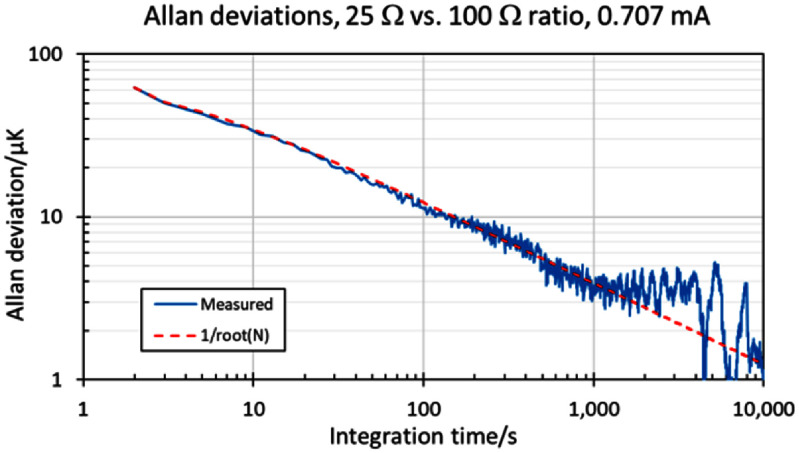
Allan deviation plot of the resistance ratio measurement of a 25 Ω standard resistor against a 100 Ω standard resistor for a current of 0.707 mA. The red dashed line represents the scaling of the Allan deviation with relation to the inverse of the square root of the number of measurements *N*.

The repeatability of the bridge used in this study was determined from the Allan deviation analysis [37] of continuous measurements of the resistance ratios of a 25 Ω standard resistor against a 100 Ω standard resistor. Figure 18 shows the Allan deviation plot of this analysis. The noise level of the measurements with the resistance bridge is of the order of 4 μK for an integration time over 1000 s (or ∼17 min), which is the value adopted as uncertainty component due to repeatability with normal distribution.

#### 5.6.2. Bridge non-linearity

The non-linearity of the bridge was determined from measurement using an automatic resistance bridge calibrator [38]. A total of 41 direct and complementary resistance ratios were measured, and the estimated non-linearity was $19.2\cdot10^{-9}$. For a 100 Ω reference resistor, this corresponds to an uncertainty contribution of 19 μK with normal distribution.

#### 5.6.3. Standard resistor

The 100 Ω reference resistor calibration is traceable to NIST’s quantum Hall realization, with an expanded (*k*= 2) uncertainty of 0.05 μΩ/Ω. It is maintained in an air bath at temperature of 25 °C controlled within ±10 mK. For a temperature-dependent resistivity coefficient *β* of $5 \cdot {10^{ - 7}}{\text{ }}{{\mathrm{K}}^{ - 1}}$, the uncertainty component due to the standard resistor is 6 μK.

##### 5.6.4. Phase transition repeatability.

The repeatability of the TP CO_2_ and TP SF_6_ realizations reported in sections 4.1 and 4.2 were determined from the analyses of full realization plateaus at 50 % of melted fraction. For this uncertainty assessment, the repeatability values were determined from the calibrations of SPRTs 0103 and 1528 at the fixed points, which were performed around 50% of melted fraction, but not exactly at that point of the plateaus, hence some degree of variability. Nonetheless, the estimated values of repeatability for the triple points of CO_2_ and SF_6_ were 36 μK and 74 μK, respectively, with normal distribution.

#### 5.6.5. Chemical impurities

As reported in section 2 and detailed in section 5.2, we assumed that the purifier installed in the manifold used for filling the cells removed all moisture, O_2_ and hydrocarbons down to the 10^−9^ level, leaving behind, for cell CO_2_ 810, only N_2_ and Ar. We also assumed that the degassing step adopted for cell CO_2_ 810 reduced both N_2_ and Ar levels down to the same as cell 811. It has been demonstrated that the estimated molar fraction of N_2_ in the liquid phase of cell 811 was 0.03 μmol mol^−1^, which was taken as the remaining impurity molar fraction and is equivalent to 1.4 μK. For a rectangular distribution, the standard uncertainty due to impurities in the CO_2_ cells was rounded up to 1 μK.

For SF_6_ 803 cell, the reported molar fraction of N_2_ in the gas sample was 1.6 μmol mol^−1^, while the fraction dissolved in the liquid phase was only 1.3 μmol mol^−1^. This is equivalent to a temperature depression of 0.1 mK. Assuming that the purifier removed all other contaminants down to negligible levels, and a rectangular distribution for the molar fraction of N_2_, the estimated uncertainty due to impurities in the SF_6_ cell was 0.059 mK.

#### 5.6.6. Gas pressure

As also detailed in section 5.2, the portion of the total molar fraction of N_2_ that was not diluted in the liquid may be responsible for an increase in the pressure of the vapor. For the TP CO_2_, this increase was estimated as 0.27 Pa, with negligible impact on the triple point temperature, so we assumed an uncertainty of 1 μK. For the TP SF_6_, the pressure increase was estimated as 11 Pa, which is equivalent to 7 μK. For a rectangular distribution, the uncertainty due to the gas pressure for the TP SF_6_ was 4 μK.

#### 5.6.7. Isotopic variation

Section 5.3 discusses in detail the analysis of isotopic variation of CO_2_ and SF_6_ samples. For CO_2_, the estimated isotopic variability ranges for ^18^O and ^13^C were 5.7 μK and 1.4 μK, respectively, with an expected uncertainty of the order of 10 %. For a rectangular probability distribution, this corresponds to an uncertainty contribution of 4 μK. For SF_6_, the estimated range was 9 μK with an uncertainty factor of ∼3. Therefore, also for a rectangular distribution, the uncertainty due to isotopic variation of SF_6_ was estimated as 16 μK.

#### 5.6.8. Hydrostatic head correction

The uncertainty of the height of the liquid column was assigned as ±1 cm for both TP CO_2_ and TP SF_6_ cells. Taking the d*T/*d*h* values of 24.7 μK cm^−1^ and 116 μK cm^−1^ and assuming a rectangular distribution, the estimated uncertainties due to the hydrostatic head correction were 14 μK and 67 μK for CO_2_ and SF_6_, respectively.

#### 5.6.9. Self-heating correction

The measurements of the full realization plateaus of the triple points of CO_2_ and SF_6_ were measured at a finite current of 0.707 mA. However, the calibration data of the SPRTs measured at all the fixed points of Ar, CO_2_, SF_6_, Hg, TPW and Ga used for the analysis of section 4.3 were corrected for self-heating. This allowed assessing the variability of the self-heating coefficients at the different fixed points, as also detailed in section 3.1, which we attribute to varying external self-heating values due to the different installation of the SPRTs in different cells. The amplitude of this range of values was 116 μK, which for a rectangular distribution corresponds to an uncertainty of 33 μK.

#### 5.6.10. Immersion error

The immersion error of the SPRTs was assessed by measuring how well the thermometers track the theoretical hydrostatic pressure line inside the cells during immersion tests, and the results of these tests are illustrated in figures 7 and 9. Any consistent deviation from the theoretical line is considered an indication of unwanted heat conduction from the bath and external environment to the thermowell. The measurement data was analyzed in accordance with current guidelines [39], and the estimated uncertainties due to immersion errors for the triple points of CO_2_ and SF_6_ were 15 μK and 73 μK, respectively.

#### 5.6.11. Slope of the plateau

As reported in sections 4.1 and 4.2, the estimated slopes of the plateaus of the TP CO_2_ and TP SF_6_ over the central 50 % portion of the melting range were 81 μK and 0.46 mK, which for a rectangular probability distribution correspond to uncertainty contributions of 47 μK and 0.266 mK, respectively. It is important to highlight that these are conservative estimates of the slope of the plateaus solely based on the measured data; after applying the progressive head correction the contributions can be reduced to 18 μK for the TP CO_2_ and 0.13 mK for the TP SF_6_.

#### 5.6.12. Extrapolation to F = 1

Table 9 shows the estimated liquidus point temperature values for the triple points of CO_2_ and SF_6_ for three different fitting models: *F, F^−^*^1^ and *F^−y^*. The values in parenthesis correspond to the statistical uncertainties with normal probability distribution. The values adopted for the temperatures of the TP CO_2_ and TP SF_6_ were the ones provided by the *F^−^*^1^ model, with 5 μK and 35 μK of uncertainty with normal distribution, respectively. These uncertainties were combined with the ones due to the determination of the melted fraction *F* (i.e. 5 %) with rectangular distribution, resulting in contributions of 5 μK for CO_2_ and 36 μK for SF_6_.

#### 5.6.13. ITS-90 realization

The estimates of the realization temperatures of the TP CO_2_ and TP SF_6_ were based on the interpolation of the ITS-90 deviation function calculated for CSPRT 0103 over subrange 4, from calibrations at the TP Ar and TP Hg. The combined (*k*= 1) uncertainties for the triple points of Ar and Hg, are 0.085 mK and 0.076 mK, respectively. The propagated *k* = 1 uncertainties at the triple points of CO_2_ and SF_6_ are then, respectively, 0.103 mK and 0.093 mK.

#### 5.6.14. SPRT non-uniqueness

This component is a combination of contributions due to types 1 and 3 NU and was determined based on the models recommended in [39]. For the TP CO_2_, the contributions from the types 1 and 3 NU were, respectively, 24 μK and 74 μK, while for the TP SF_6_ these contributions were estimated as 13 μK and 53 μK, which were combined in quadrature to obtain the values of 78 μK for CO_2_ and 55 μV for SF_6_.

#### 5.6.15. TPW propagation

The combined (*k*= 1) uncertainty of the TPW measurements was 42 μK and were propagated to the triple points of CO_2_ and SF_6_ using factors of 0.745 and 0.774, respectively, resulting in uncertainty contributions of 31 μK and 32 μK.

### 5.7. Suitability of the TP CO_2_ and the TP SF_6_ as fixed points in a future revision of the ITS-90

The results of measurements of the TP CO_2_ and TP SF_6_ cells studied in this work demonstrated that it is possible to realize these fixed points with reproducibility ∼<0.1 mK, and with estimated temperatures in agreement with the reported values in the literature. Such level of reproducibility observed, especially with the TP CO_2_ cells, was achieved even for cells manufactured 6 years apart using gas samples from different suppliers. This is of the same order of magnitude as the reproducibility of TP Hg cells, which qualifies the TP CO_2_ as a viable replacement for mercury on the ITS-90, provided that a consensus value for its realization temperature is finally reached.

**Figure 19.**
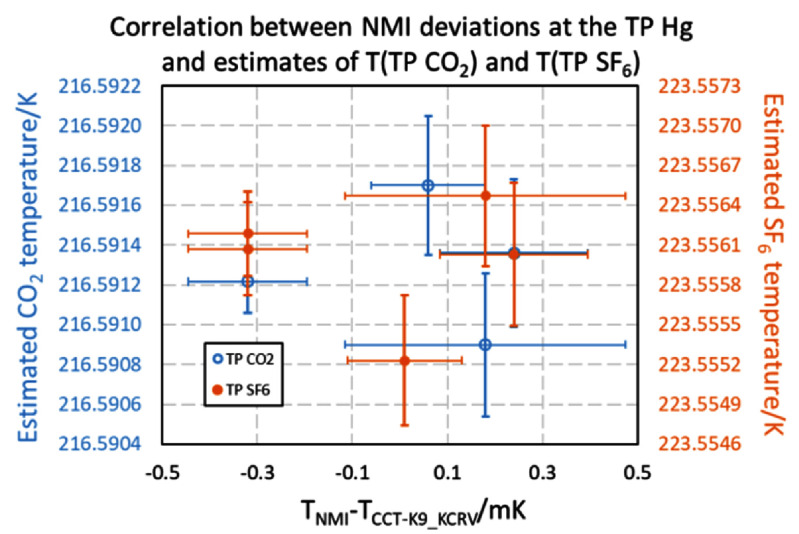
Correlation between the NMI deviations at the mercury triple point and their temperature estimates for the triple points of CO_2_ (open blue circles) and SF_6_ (solid orange circles). Error bars correspond to the combined uncertainties (*k* = 1) of the plotted values.

It is important to highlight again that the results above were obtained for temperature values and uncertainties that derived from literature data, as well as for the data obtained in this study. This means that the nominal assigned values for the TP CO_2_ of 216.591 40(49) K and for the TP SF_6_ of 223.556 00(65) K can be considered good approximations to *T*_90_ consensus values of these fixed points. The estimated realization temperatures of the triple points of CO_2_ and SF_6_ as reported by each NMI, and their respective uncertainties, are mostly dependent on the realization temperature of their reference TP Hg cells. Figure 19, however, shows that there is no obvious correlation between NMI deviations at the TP Hg relative to the CCT-K9 key comparison reference value (KCRV) [40] and their temperature estimates for the triple points of CO_2_ and/or SF_6_, meaning that these values might be influenced by other effects than systematic errors in the mercury triple point realizations.

The results of the measurements with the two SPRTs allow a deeper analysis of the suitability of the TP CO_2_ and the TP SF_6_ as alternative fixed points to the TP Hg in a future revision of the ITS-90. To do so, we estimated the propagation of uncertainty (PoU) and the NU (although limited to the two SPRTs 0103 and 1528 used in this work), assuming that a future revision of the ITS-90 would allow the use of the triple points of CO_2_ and SF_6_ to generate alternative subranges with identical deviation functions by assigning them stipulated ITS-90 temperature values (in the same way the Hg fixed point is currently assigned the ITS-90 value of 234.3156 K).

In such case, the current subrange 4 (TP Ar, TP Hg and TPW) would have two alternative subranges, which for convenience we refer as 4*a* (TP Ar, TP CO_2_ and TPW) and 4*b* (TP Ar, TP SF_6_ and TPW). Similarly, subrange 5 (TP Hg, TPW and MP Ga) would have two alternative subranges, which we label as 5*a* (TP CO_2_, TPW and MP Ga) and 5*b* (TP SF_6_, TPW and MP Ga).

For this investigation, the values assigned for the temperatures of the triple points of CO_2_ and SF_6_ are the same as those used in section 4.3, i.e. the averages of the values reported in tables 4 and 5. However, in this case their respective uncertainties are the combined uncertainties of table 10, excluding the components due to the extrapolation to *F*= 1 and due to scale interpolation and NU. This results in uncertainties of the TP CO_2_ and TP SF_6_ of 0.07 mK and 0.20 mK, respectively (*k*= 1), and these represent the estimated uncertainties of the realization of ITS-90 defined fixed points. Table 11 summarizes the values used for this investigation, which will assess the PoU and types 1 and 3 NU.

**Table 11.** Temperatures and uncertainties adopted for PoU and non-uniqueness investigation.

Subrange	Fixed points			Fixed point realization uncertainty (*k* = 1)/mK			Assigned ITS-90 temperature/K		
	FP1	FP2	FP3	FP1	FP2	FP3	FP1	FP2	FP3
4	TP Ar	TP Hg	TPW	0.085	0.076	0.042	83.805 8	234.315 6	273.16
4*a*	TP Ar	TP CO_2_	TPW	0.085	0.068	0.042	83.805 8	216.591 40	273.16
4*b*	TP Ar	TP SF_6_	TPW	0.085	0.196	0.042	83.805 8	223.556 00	273.16
5	TP Hg	TPW	MP Ga	0.076	0.042	0.065	234.315 6	273.16	302.914 6
5*a*	TP CO_2_	TPW	MP Ga	0.068	0.042	0.065	216.591 40	273.16	302.914 6
5*b*	TP SF_6_	TPW	MP Ga	0.196	0.042	0.065	223.556 00	273.16	302.914 6

#### 5.7.1. PoU

**Figure 20.**
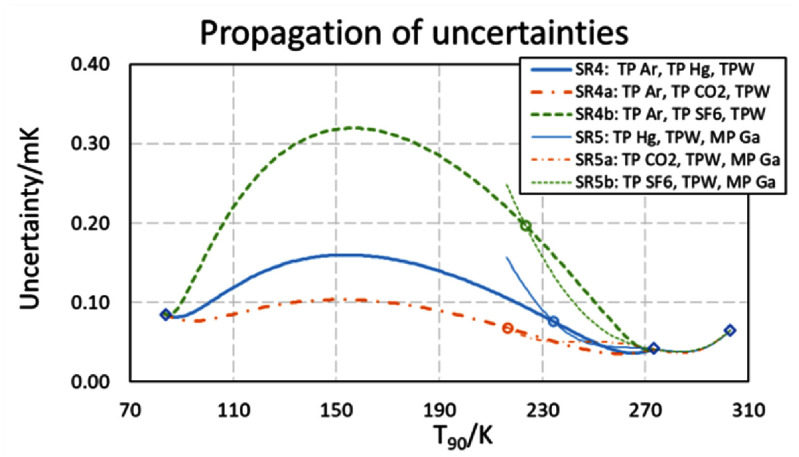
Propagation of uncertainties over the ITS-90 subranges from the TP Ar to the TPW and from the TP Hg to the MP Ga using either the triple points of Hg, CO_2_ or SF_6_. Circles correspond to the uncertainties (*k*= 1) at the TP Hg, TP CO_2_ and TP SF6, and diamonds correspond to the uncertainties at the TP Ar, TPW and MP Ga.

Figure 20 illustrates the PoU over subranges 4 and 5 using either the TP Hg, TP CO_2_ or TP SF_6_. For subrange 4, the more centralized position of the triple point of CO_2_ results in smaller interpolation uncertainties over the full range, while the still relatively high uncertainty of the TP SF_6_ results in higher interpolation uncertainties. For subrange 5, although the TP Hg provides a slightly lower uncertainty on a limited interval of the range, as the interpolated temperatures get lower, the uncertainties using the TP CO_2_ become lower than the ones obtained using the TP Hg. Additionally, the subrange interpolation 5a covers a wider temperature range with reduced uncertainties, while the others would provide larger uncertainties due to extrapolation if traceability down to 216 K is required—it is not unusual that calibration customers request extrapolations from the TP Hg down to −50 °C or −60 °C, which result in larger uncertainties. In summary, with the current realization uncertainties, the TP CO_2_ is certainly the more advantageous fixed point in terms of interpolated uncertainties in both subranges.

#### 5.7.2. Subrange inconsistency (SRI)

**Figure 21.**
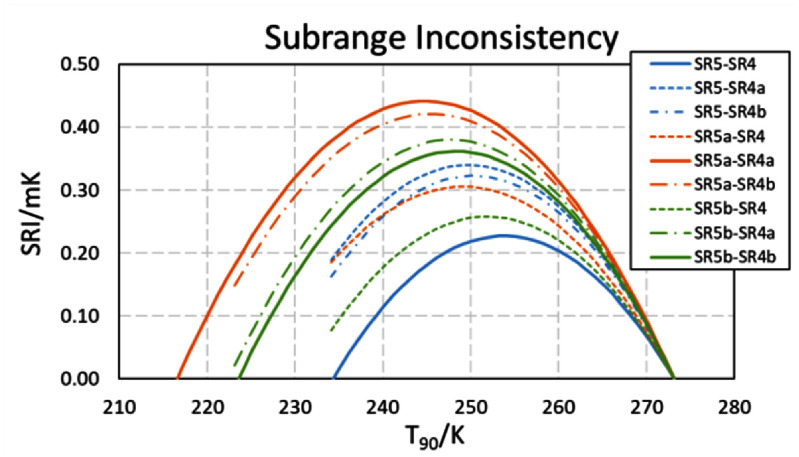
Subrange Inconsistency (SRI) analysis of CSPRT 0103, showing the differences between subranges 4 and 5 using measured values at either the triple points of Hg (SR5–SR4), CO_2_ (SR5a–SR4a) or SF_6_ (SR5b–SR4b). Solid lines correspond to the SRI estimated using the same fixed point for both subranges 4 and 5, while the dashed lines correspond to different combinations of fixed points in each subrange.

Also known as type 1 NU [39], it arises from the differences between the interpolated values using the deviation functions of different subranges. For the scope of this work, the SRI under analysis corresponds to the differences between the interpolated values obtained from subranges 4 and 5 only. Figure 21 illustrates the SRI over the temperature range where the two subranges overlap, as generated by the calibration data of the CSPRT 0103. The analysis includes the cases where the same fixed point (either TP Hg, TP CO_2_ or TP SF_6_) is used for determining both subranges and also the cases where different fixed points are used for each subrange. It can be concluded that, regardless of the combination of fixed points used in each subrange, the SRI magnitude scales with the width of the overlapping temperature range. The higher SRI values for subranges 4a, 4b, 5a and 5b could also be explained partially by the differences between the average fixed-point temperatures adopted for this analysis and the estimated temperatures of the fixed points at F = 0.5, i.e. the estimated temperatures at which the SPRTs were calibrated, and partially by the natural measurement errors at the fixed points, which are already accounted for and propagated into the interpolated uncertainties, as also reported by [41].

#### 5.7.3. Intra-subrange inconsistency (*Intra-SRI*)

The definition of SRI establishes that it corresponds to the differences between the interpolated temperature values using deviation functions of overlapping ITS-90 SPRT subranges [39]. According to the scale definition, each subrange is defined by calibrating a SPRT at a specific set of fixed points, and it does not predict the use of alternative or redundant fixed points within the same subrange. Therefore, the adoption of the triple points of CO_2_ and SF_6_ as alternatives to the triple point of mercury creates a new kind of inconsistency which here is called *Intra-SRI* and accounts for the differences between the interpolations of the same subrange calculated by different combinations of fixed points.

**Figure 22.**
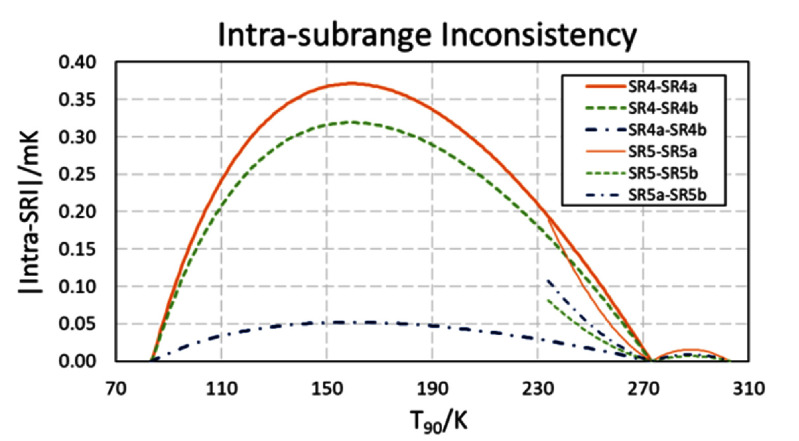
Intra-subrange inconsistency (Intra-SRI) analysis of CSPRT 0103, showing the absolute differences between the same subrange obtained using measured values at either the triple points of Hg, CO_2_ or SF_6_. Solid lines represent the analysis of subrange 4 and dashed lines represent the analysis of subrange 5.

Figure 22 illustrates the Intra-SRI analysis obtained from the calibration data of CSPRT 0103 for both subranges 4 (and 4*a* and 4*b*) and 5 (and 5*a* and 5*b*). It can be seen that the Intra-SRI is significantly higher at temperatures below the TPW than above, which can be explained by the higher sensitivities with relation to the TPW and the MP Ga above 273.16 K. In this case, differently from the traditional SRI, the magnitudes of the Intra-SRI are mostly justified by the differences between the average fixed-point temperatures used for the analysis and the estimated temperatures at the TP CO_2_ and TP SF_6_ at *F*= 0.5, at which the thermometers were actually calibrated. To provide a notion of the impact of these differences, the Intra-SRI for CSPRT 0103 was also evaluated for the case where the assigned fixed-point temperatures for the triple points of CO_2_ and SF_6_ were the ones estimated for *F* = 0.5, and its magnitude dropped to less than 0.15 mK for subrange 4, about the same magnitude of the propagated uncertainties. This means that the Intra-SRI analysis will be a useful tool for estimating consensus values for the triple points of carbon dioxide and sulfur hexafluoride in a future revision of the scale.

#### 5.7.4. Type 3 non-uniqueness

Type 3 NU is observed by obtaining different interpolated values for the same measured temperature when using different SPRTs calibrated on the same subrange [39]. In this study, this can be investigated by comparing the interpolated values of SPRTs 0103 and 1528 at the TP CO_2_ and TP SF_6_. Both thermometers were calibrated at the same melted fraction (around *F*= 0.5) of all fixed points, so it is reasonable to assume that such measurements can be regarded as simultaneous isothermal measurements of the two SPRTs at the fixed points and that the interpolated values from their calibrations should be the same. Table 12 provides the estimates of type 3 NU, obtained from the differences between the interpolated values of SPRTs 0103 and 1528 at the fixed points over subranges 4, 4*a* and 4*b*. For all fixed points and subranges, the values of NU were always lower than the measurement uncertainties.

**Table 12.** Type 3 non-uniqueness analysis. Values in millikelvin.

	Subrange		
Fixed point	4	4*a*	4*b*
TP Hg	—	0.001	−0.057
TP CO_2_	−0.001	_—_	−0.078
TP SF_6_	0.070	0.071	—

### 5.8. Reaching consensus values for the triple points of CO_2_ and SF6

The final report of the key comparison CCT-K9 allows analyzing the spread of the results of the participant NMIs at the TP Hg [40]. The amplitude of the spread of the reported TP Hg differences with relation to the CCT-K9 KCRV for the NMIs that contributed with estimates of the temperatures of the triple points of CO_2_ and SF_6_ is of the order of 0.5 mK. Differences in the realized TP Hg temperatures, if assumed as systematic errors, propagate to the TP CO_2_ and SF_6_ with factors of 1.36 and 1.23, meaning that the spread at the mercury point measurements may increase up to 0.68 mK and 0.62 mK, respectively. The estimated temperatures from the literature in tables 4 and 5 show spreads of the order of 1 mK, which in principle could be due to systematic errors propagated from their TP Hg realizations. However, figure 18 suggests that there is no correlation between the TP Hg realization and the estimates of the triple points of CO_2_ and SF_6_, meaning that other factors than the TP Hg realizations are impacting their estimates. These might include, among other factors, type 3 NU and the presence of impurities in the gas samples.

The selection criteria that we applied to obtain the CO_2_ and SF_6_ TP realization temperatures shown in tables 4 and 5 were as follows. (a) The work is contemporaneous, limited to the last 10 years. (b) The uncertainties were all comparable and ∼<0.5 mK. (c) The publications provided sufficient detail to evaluate and compare the uncertainties. This resulted in our omitting the many CO_2_ TP results listed in Pavese and Steur [42] which were much older, the most recent realization being from 2001. Those more recent realizations were unpublished and only one of the CO_2_ cells with results reported there, a cell identified as ‘CO_2_-9’, had a reported uncertainty of ⩽0.5 mK. If the reported realization temperature for CO_2_-9 from 2001 was included into our table 4, the revised average CO_2_ TP value would be 216.591 46(48) K.

One way of determining consensus values for the triple points of CO_2_ and SF_6_ could be a comparison between the NMIs that possess such cells (or intend to in the near future) over subrange 4 of the ITS-90, between the TP Ar and the TPW. In this comparison, SPRTs would be calibrated at the triple points of Ar, SF_6_, CO_2_ and Hg. The experience with the CCT-K9 demonstrated the difficulties associated with the shipping of LSPRTs, reflected in drifts and dark uncertainty. The shipping of triple point cells involves the regulatory restrictions on the of transport of high pressure compressed gases. Therefore, the best option for such comparison would be to utilize CSPRTs as the traveling standards. Despite being as fragile as LSPRTs, CSPRTs have the advantage of being small and easy to hand carry by courier, and are very stable as long as they are handled with the proper care. We recently demonstrated this approach in a regional key comparison of the Ar TP [43]. In the case of CO_2_ and SF_6_, a set of at least two CSPRTs would travel to the participant NMIs, being calibrated at all the available fixed points between the triple points of argon and the TPW. This data set would allow estimating the temperatures of the triple points of CO_2_ and SF_6_ with greater accuracy than the averaged estimates presented in this work.

## 6. Conclusions

This work described the manufacture and assessment of two immersion-type TP CO_2_ cells and one immersion-type TP SF_6_ cell. These cells were measured multiple times with two calibrated SPRTs, and the estimated *T*_90_ values were 216.591 19(15) K at *F*= 0.5 and 216.591 21(15) K at *F* = 1 for the TP CO_2_, and 223.556 12(33) K for *F* = 0.5 and 223.556 22(33) K at *F* = 1 for the TP SF_6_. The reported uncertainties are for a coverage factor *k* = 1. These values are in agreement with the ones reported in the literature.

It was demonstrated that the reproducibility of <0.1 mK for these fixed points is achievable, but is also dependent on the realization technique. It was shown that a controlled inner freeze method using immersion coolers, carefully filled with a known mass of dry ice powder to obtain cylindrical solid mantles before the realization, significantly improved the slope and the reproducibility of the plateaus. The technique was proposed based on the contraction of both CO_2_ and SF_6_ as they freeze, which impacts the shape of the solid mantle depending on the freeze method. The expansion of CO_2_ and SF_6_ upon melt is also responsible for a progressive head correction that results in additional slopes on the realizations plateaus that can be corrected for with prior knowledge about the cell characteristics and the realization method. The impact of this effect was larger than any other effect on the slopes of the plateaus, especially for the TP CO_2_. This effect can only be observed in large immersion-type cells, and are expected to be negligible when using small adiabatic cells.

The reproducibility of the realizations of the TP CO_2_ was better than 25 μK, and the slope of the plateaus was 80 μK on average over 50 % of melted fraction, with 50 μK of this slope due to the progressive head effect that can be corrected. This accuracy level is comparable to that of TP Hg cells, which finally qualifies the triple point of CO_2_ as a suitable alternative for the mercury point in the ITS-90, if all cares are taken during the realization. The TP SF_6_, although less accurate (mostly due to a lower sample purity), remains a promising alternative to the mercury point as well, with a reproducibility better than 75 μK and an average slope of 0.46 mK over 50 % of the melted fraction, with 0.23 mK of the slope due to the progressive head correction.

The cells manufactured for this study were used to calibrate two SPRTs on subranges 4 (TP Ar to TPW) and 5 (TP Hg to MP Ga) of the ITS-90 using TP Hg, TP CO_2_ and TP SF_6_ cells. The results showed that the agreement between the interpolated values obtained using either Hg, CO_2_ or SF_6_ triple point cells and the measured values in the cells was always better than the combined *k*= 1 uncertainties. It is important to emphasize that the CSPRT 0103 was adapted for use as a long-stem glass-sheathed thermometer with similar immersion characteristics to those of a conventional LSPRTs. This in practice validates the use of TP CO_2_ and TP SF6 cells for regular ITS-90 calibrations of LSPRTs, if the cells are properly characterized. These results allowed for the investigation of the suitability of the triple points of CO_2_ and SF_6_ to be included in a future revision of the ITS-90 in terms of PoU, inter- and intra-SRI, and type 3 NU. While the PoU and type 3 NU analyses strongly support the adoption of the TP CO_2_ and TP SF_6_ as reference fixed points, the SRI analysis suggests that a consensus value is still needed for these points, which could be obtained by a comparison between the NMIs that detain such fixed point cells.
